# A Machine Vision Perspective on Droplet‐Based Microfluidics

**DOI:** 10.1002/advs.202413146

**Published:** 2025-01-01

**Authors:** Ji‐Xiang Wang, Hongmei Wang, Huang Lai, Frank X. Liu, Binbin Cui, Wei Yu, Yufeng Mao, Mo Yang, Shuhuai Yao

**Affiliations:** ^1^ Institute of Optics and Electronics Chinese Academy of Sciences Chengdu 610209 P. R. China; ^2^ Hebei Key Laboratory of Man‐Machine Environmental Thermal Control Technology and Equipment Hebei Vocational University of Technology and Engineering Hebei 054000 China; ^3^ Department of Mechanical and Aerospace Engineering The Hong Kong University of Science and Technology Clear Water Bay, Kowloon Hong Kong SAR 999077 China; ^4^ School of Science and Technology Hong Kong Metropolitan University Ho Man Tin Hong Kong SAR 999077 China; ^5^ Department of Biomedical Engineering The Hong Kong Polytechnic University Hung Hom, Kowloon Hong Kong SAR 999077 China; ^6^ Department of Chemical and Biological Engineering The Hong Kong University of Science and Technology Clear Water Bay, Kowloon Hong Kong SAR 999077 China; ^7^ College of Electrical Energy and Power Engineering Yangzhou University Yangzhou 225009 China; ^8^ National Key Laboratory of Optical Field Manipulation Science and Technology Chinese Academy of Sciences Chengdu 610209 China

**Keywords:** artificial intelligence, data‐driven automation, intelligent multiphase flows, label‐free method, microfluidic droplets

## Abstract

Microfluidic droplets, with their unique properties and broad applications, are essential in in chemical, biological, and materials synthesis research. Despite the flourishing studies on artificial intelligence‐accelerated microfluidics, most research efforts have focused on the upstream design phase of microfluidic systems. Generating user‐desired microfluidic droplets still remains laborious, inefficient, and time‐consuming. To address the long‐standing challenges associated with the accurate and efficient identification, sorting, and analysis of the morphology and generation rate of single and double emulsion droplets, a novel machine vision approach utilizing the deformable detection transformer (DETR) algorithm is proposed. This method enables rapid and precise detection (detection relative error < 4% and precision > 94%) across various scales and scenarios, including real‐world and simulated environments. Microfluidic droplets identification and analysis (MDIA), a web‐based tool powered by Deformable DETR, which supports transfer learning to enhance accuracy in specific user scenarios is developed. MDIA characterizes droplets by diameter, number, frequency, and other parameters. As more training data are added by other users, MDIA's capability and universality expand, contributing to a comprehensive database for droplet microfluidics. The work highlights the potential of artificial intelligence in advancing microfluidic droplet regulation, fabrication, label‐free sorting, and analysis, accelerating biochemical sciences and materials synthesis engineering.

## Graphical abstract

A machine vision approach using Deformable detection transformer is developed for accurate identification, sorting, and analysis of microfluidic droplets as well as encapsulated cells. Possessing transfer learning functionality, the established online software MDIA can serve as a powerful in situ real‐time tool for monitoring, classifying, and controlling microfluidic droplets, supporting label‐free sorting, tracing, automatic statistical analysis, and automated microfluidic experimentation.

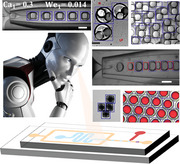

## 1 Introduction

Microfluidics, a multidisciplinary field at the forefront of innovation, leverages precise manipulation of fluids at the microscale to revolutionize chemical, biological, and materials synthesis research.^[^ ^1^, ^2^, ^3^, ^4^, ^5^ ^]^ Droplet microfluidics enables highly parallelized and miniaturized tests by consistently distributing liquid samples, from nanoliters to picoliters, within a nonmixing carrier liquid.^[^ ^6^ ^]^ Microdroplets, the fundamental units, offer unique features such as isolated compartments for single‐cell or single‐molecule assays, rapid mixing, negligible thermal inertia, and the presence of immiscible liquids for novel material synthesis and interface reactions.^[^ ^7^ ^]^ Controlled microfluidic systems facilitate high‐throughput assays,^[^ ^8^, ^9^ ^]^ enabling intricate reactions,^[^ ^3^ ^]^ targeted drug loading and delivery,^[^ ^10^ ^]^ single‐cell studies,^[^ ^11^ ^]^ advanced manufacturing,^[^ ^12^, ^13^ ^]^ atomic energy conversion,^[^ ^14^ ^]^ and droplet cooling system.^[^ ^15^, ^16^ ^]^ This highlights the pivotal role of microdroplet technology in miniaturization, efficiency, and transformative applications, from disease diagnosis to environmental monitoring and advanced material synthesis.

In microfluidics, precise control over the morphology and generation rate of microdroplets is crucial.^[^ ^17^ ^]^ Microfluidics primarily generates numerous compartments for high‐throughput screening^[^ ^18^ ^]^ and the synthesis of functional materials composed of millions of droplets.^[^ ^19^ ^]^ The generation rate significantly impacts application efficiency,^[^ ^20^ ^]^ while droplet size influences reaction kinetics and thermodynamics.^[^ ^21^ ^]^ Smaller droplets, with their higher surface‐to‐volume ratio, enhance heat and mass transfer, accelerating reactions—essential for biological quantification^[^ ^22^, ^23^, ^24^ ^]^ and chemical synthesis.^[^ ^25^ ^]^ Uniform droplet size ensures replicable results in high‐throughput screening, such as drug discovery or biocatalyst testing, which is critical for statistical significance.^[^ ^26^ ^]^ In liposome‐mediated drug delivery, droplet size affects system effectiveness.^[^ ^27^ ^]^ For material synthesis, such as Janus^[^ ^31^ ^]^ and core–shell droplets,^[^ ^32^ ^]^ size controls layer thickness and core/shell ratios, impacting encapsulation efficiency and material properties.^[^ ^33^, ^34^, ^35^, ^36^ ^]^

Besides the significance of generation rate and size, a general effective morphology of a microfluidic droplet is also critical. For instance, in single‐cell applications, only the droplets that encapsulate a single cell are considered effective, whereas those containing multiple cells or no cells are deemed ineffective. However, in certain other contexts, droplets are considered effective as long as they contain cells, regardless of the number of cells present.^[^ ^33^ ^]^ For the application of double emulsions (DEs), in some occasions, only DEs with a single core are preferred for exact controlled environment, quantitative nutrition supply, and reagent diffusion necessary for cell viability or molecular analysis.^[^ ^28^, ^29^ ^]^ In other applications, multicore DEs are more suitable.^[^ ^30^ ^]^ Additionally, the concentricity of DEs is critically important as it ensures precise control over droplet stability, enabling accurate encapsulation, controlled mass transfer, cellular mimicry, and advanced material synthesis.^[^ ^32^, ^35^, ^37^ ^]^

Traditionally, creating microfluidic droplets with specific user‐preferred morphology has been a laborious and resource‐intensive process.^[^ ^38^ ^]^ For example, enzyme labeled method is a prevailing method for selecting droplets with desired cell‐encapsulated droplets. However, such a method is not only labor‐intensive and inefficient, but the newly introduced markers may also potentially disrupt the intrinsic properties. Therefore, a label‐free approach is highly demanded.^[^ ^39^, ^40^ ^]^ Recently, machine learning (ML) has emerged as an effective method for predicting microfluidic droplets with user‐desired size and frequency. Lashkaripour et al. present DAFD, a web‐based tool that leverages ML to automate the design of flow‐focusing microfluidic devices for microfluidic single droplet (SD) and DE generation.^[^ ^38^, ^41^ ^]^ DAFD employs ML algorithms to predict droplet diameter and generation rate with high accuracy, reducing errors from 4.2% to 11.5% of the specified diameter and rate. Wang et al. proposed a dual‐directional deep learning algorithm to acquire coflowing microfluidic droplets with the average relative error of 9.90%.^[^ ^42^ ^]^ Despite these advances, there are limitations. Current statistical ML methods can only generate single and DE droplets with moderate precision, typically around 10% relative error.^[^ ^38^, ^41^, ^42^, ^43^ ^]^ Additionally, ML models are often tailored to specific chip designs; for example, DAFD is effective for flow‐focusing chips^[^ ^38^, ^41^ ^]^ but not for other types like cofocusing or T‐junctions, while Wang's model is limited to cofocusing devices.^[^ ^42^ ^]^ In summary, existing ML approaches are mainly used in the initial design phase of microfluidic devices and operating condition selection, which results in droplet morphology close to, but not exactly matching, the target. This requires additional manual fine‐tuning. Moreover, monitoring other important droplet properties, such as frequency, core/shell ratio, and concentricity, still demands significant human effort.

To eliminate the need for high labor‐intensity, frequent manual adjustments, and achieve fully autonomous microfluidic laboratories,^[^ ^44^, ^45^ ^]^ it is prerequisite to introduce downstream automatic monitoring, identification, and sorting systems that can replace human labor. Machine vision recognition has the potential to serve as an integral component of such an automated monitoring system, effectively bridging upstream design and downstream fabrication processes. Recently, some basic machine vision algorithms such as edge detection, Hough transformer, and you only look once (YOLO) have been applied to the identify microfluidic SDs.^[^ ^42^, ^46^, ^47^, ^48^, ^49^, ^50^ ^]^ However, such simple machine vision algorithms can be hardly applied to identify and detect DE droplets as demonstrated in Movie S1 (Supporting Information). Thus, more advanced machine vision algorithms are required to fulfill identification of single and DE droplets or even cell‐encapsulated droplets.

Most recently, Faster R‐CNN^[^ ^51^ ^]^ and Deformable detection transformer (DETR)^[^ ^52^ ^]^ was developed with improved training efficiency and detection accuracy. Unlike object detection models that require separate stages for training (e.g., proposal generation, bounding box regression, and mask prediction), Deformable DETR instead of Faster R‐CNN can be trained end‐to‐end. This means that the entire model, including the backbone, transformer, and prediction heads, is trained jointly with a single objective, simplifying the training data and training process.^[^ ^53^ ^]^ In addition, compared with Faster R‐CNN, the deformable attention module and multiscale feature integration in Deformable DETR are responsible for improved performance in small objects detection,^[^ ^52^ ^]^ which is more suitable for both microfluidic droplet and cell detections. Thus, Deformable DETR has great potential to enable image‐based label‐free droplet sorting in cell‐involved microfluidic applications.

**Figure 1:**
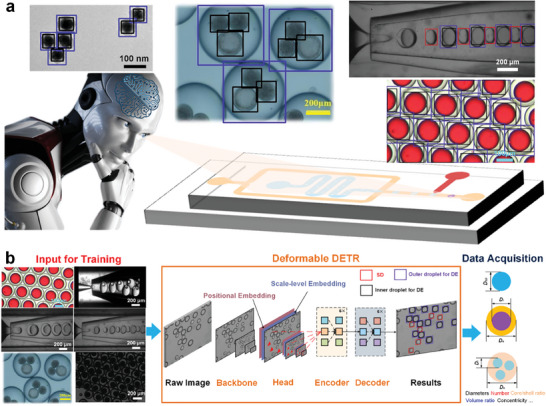
Conception of the machine vision on Microfluidic droplets. a) An overview of this study. The trained machine vision can identify both single and DE droplets, as well as other core–shell structures. The original images here for identification demonstration come from Liang's,^[^ ^54^ ^]^ Zhang's,^[^ ^60^ ^]^ Mettler's,^[^ ^56^ ^]^ and our experimental works. Reproduced with permission.^[^ ^54^ ^]^ Copyright 2024, Wiley. Reproduced with permission.^[^ ^60^ ^]^ Copyright 2018, American Institute of Physics. b) Machine vision framework consisting of training, object detection, and data processing modules. High‐resolution images are acquired using high‐speed optical imaging to generate a large input image dataset for training the Deformable DETR. After training, raw images are delivered into the Deformable DETR module where the microfluidic droplets in the raw images can be identified. The detected data then, undergoes data processing module where useful parameters of the detected droplets can be obtained.

Herein, we leverage this newly‐developed machine vision algorithm—Deformable DETR—to rapidly detect multiscale single and DE droplets generated from multiple microfluidic systems and scenarios. Exploiting the data from our experiments and previous publications, we present a new machine vision methodology, MDIA (Microfluidic Droplets Identification and Analysis), a web‐based tool that can detect microfluidic droplets and analyze their characteristics such as diameter, number, frequency, as well as core/shell ratio and concentricity for DEs according to the identification results. An overall conception of this study is shown in **Figure** **1**a. For the purpose of universality, this online and open‐source web‐based tool (MDIA) also supports transfer learning, allowing users to upload their own microfluidic droplet image data for training and acquire microfluidic droplet identification with higher fidelity and accuracy for their own specific scenarios. Strategies for optimizing the transfer learning process are also offered in this study.

## 2 Results

### 2.1 Establishment of the Proposed Deformable DETR

Figure 1b demonstrates the schematic view of the utilized Deformable DETR framework composed of four components: Backbone, Head, Encoder, and Decoder. The Backbone is primarily utilized to extract multiscale image feature maps (MSFMs) of microfluidic droplets, facilitating subsequent feature integration. The Head receives features transmitted from the Backbone and constructs positional embeddings and scale‐level embeddings. With these embeddings, enhanced MSFMs are formed, serving as inputs to the subsequent Encoder. The primary function of the Encoder section is to learn the global features of microfluidic droplet images across different scales using the self‐attention mechanism. Afterward, the Decoder is responsible for transforming the feature information generated by the Encoder into recognizable target positions and class information, thereby achieving object recognition and localization. As demonstrated in Figure 1b, there are three class labels: 1) SD, 2) outer droplet, and 3) inner droplet(s) for DE droplets. Please refer to Note S1 (Supporting Information) for detailed operation mechanism of the Deformable DETR.

**Figure 2:**
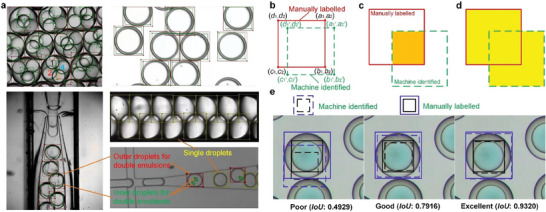
Schematics of manually labeled and machine identified microfluidic droplets and evaluation system. a) Typical manually labeled microfluidic droplets for training and evaluation. Microfluidic droplet images for various scenarios such as SDs and DE droplets (single inner droplet and multiple inner droplets) were adopted for manual annotation with rectangular manually labeled boxes for wide applications of microfluidic droplet research and industry. b) Rectangular schematic manually labeled, and machine identified boxes with coordinate information. c) Intersection area (filled by orange) and d) Union area (filled by yellow) between the manually labeled and machine identified boxes. e) examples of poor, good, and excellent identification with *IoU* values.

After the establishment of the Deformable DETR, parameter tuning using the training and testing dataset with manually labeled microfluidic droplets was conducted to optimize the machine vision model. Typical images of the manually labeled microfluidic droplets with multiple scenarios are displayed in **Figure** **2**a. The manually labeled rectangular boxes (MLRBs) with human annotations were utilized to train the Deformable DETR model, enabling it to comprehend the tangible forms of microfluidic droplets and associate different morphologies with their respective microfluidic droplet classifications. There are three annotated classifications as shown in Figure 2a, namely SDs, outer droplets for DE droplets, and inner droplets for DE droplets. Parameters (learning rate and epoch) tuning results are based on the comparisons between MLRBs (which are regarded as ground truth boxes) and machine identified boxes (MIB) in the testing dataset. Quantitatively, three evaluation indicators, namely length error δ_l_ (calculated by Equation (1)), area error δ_A_ (calculated by Equation (2)), and intersection over union (*IoU*) (calculated by Equation (3)), were adopted to select the optimized parameters for the excellent identification, as shown in the right‐hand side of Figure 2e with a higher *IoU* (>0.93). As displayed in Figure 2e, an excellent identification usually has a higher *IoU* with simultaneously smaller δ_l_ and δ_A_. The selected optimized learning rate and epoch are 3e‐4 and 80, respectively, achieving constant excellent identifications (δ_l_ = 4.3%, δ_A_ = 4.0%, and *IoU* = 92.5%). Please refer to Note S2 (Supporting Information) for the detailed tuning process and quantitative results

(1) $$\delta l=\frac{\sqrt{{\left(d_{1}-{d^{\prime }}_{1}\right)}^{2}+{\left(d_{2}-{d^{\prime }}_{2}\right)}^{2}}+\sqrt{{\left(b_{1}-{b^{\prime }}_{1}\right)}^{2}+{\left(b_{2}-{b^{\prime }}_{2}\right)}^{2}}}{\sqrt{{\left(d_{1}-b_{1}\right)}^{2}+{\left(d_{2}-b_{2}\right)}^{2}}}$$

(2) $$\delta_{A}=\frac{\left|\left|\left(b_{1}-d_{1}\right)\left(b_{2}-d_{2}\right)\right|-\left|\left({b^{\prime }}_{1}-{d^{\prime }}_{1}\right)\left({b^{\prime }}_{2}-{d^{\prime }}_{2}\right)\right|\right|}{\left|\left(b_{1}-d_{1}\right)\left(b_{2}-d_{2}\right)\right|}$$

(3) $$IoU=A_{\mathrm{orange}}/A_{\mathrm{yellow}}$$

where *d*_1_, *d*_2_, *b*_1_, and *b*_2_ are coordinates of the MLRB as shown in Figure 2b; *d*′_1_, *d*′_2_, *b*′_1_, and *b*′_2_ are coordinates of the MIB as also shown in Figure 2b; *A*_orange_ is the area of intersection between the MLRB and MIB as demonstrated in Figure 2c; and *A*_yellow_ is the area of union between the MLRB and MIB as demonstrated in Figure 2d.

### 2.2 Microfluidic Droplet Generation Platforms and Model Training

To guarantee a high universality, we built various microfluidic droplet generation platforms and constructed a general base of the training dataset. Note S3 (Supporting Information) details our five established microfluidic systems for generating multiple microfluidic droplets including normal oil–water–oil (o–w–o) DEs, nanoberries^[^ ^55^ ^]^ encapsulated liposomal DEs, SDs, phase change material (PCM) encapsulated DEs, cell encapsulated droplets. Please refer to the Experimental Section for detailed fabrications of these microfluidic systems. Based on the established microfluidic platforms, multiple microfluidic droplet generation images and movies were obtained. Some of the microfluidic droplet visualization results from our experimental platforms were used for microfluidic droplet manual labeling, acting as a major part in the training and testing of data for optimizing parameters of the Deformable DETR (see Note S2, Supporting Information). After optimization, the model was trained by a set of training data (see the Experimental Section). The microfluidic droplet visualization results from the platform in Figures S3 and S5 in Note S3 (Supporting Information) were used for the validation dataset of the Deformable DETR for identification demonstrations.

### 2.3 Microfluidic Droplet Identification and Analysis

#### 2.3.1 Single‐Image‐Based Identification

**Figure 3:**
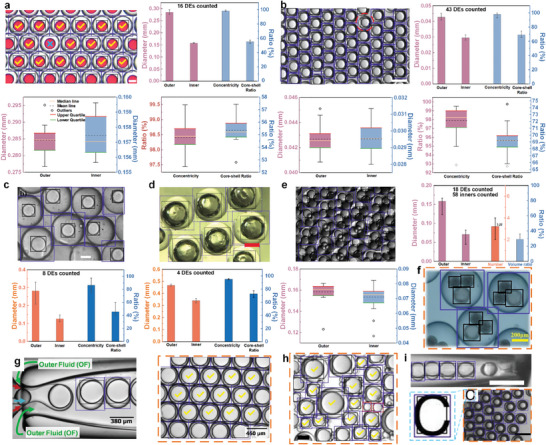
Single‐image‐based Identification of microfluidic droplets and analysis from multiple scenarios. a–i) Machine identification of the DE droplets with inner cores and statistical analyses based on the identification results. The DE droplet images in (a, b, c, d, e, f, g, h, and i) are originally from Metter's,^[^ ^56^ ^]^ Lashkaripour's,^[^ ^41^ ^]^ Kim's,^[^ ^57^ ^]^ Fu's,^[^ ^58^ ^]^ Hughes's,^[^ ^59^ ^]^ Zhang's,^[^ ^60^ ^]^ Foster's,^[^ ^61^ ^]^ Zarzar's,^[^ ^62^ ^]^ and Zhang's^[^ ^63^ ^]^ works, respectively. Reproduced with permission.^[^ ^41^ ^]^ Copyright 2024, Springer Nature. Reproduced with permission.^[^ ^58^ ^]^ Copyright 2014, Elsevier. Reproduced with permission.^[^ ^59^ ^]^ Copyright 2013, Elsevier. Reproduced with permission.^[^ ^60^ ^]^ Copyright 2018, American Institute of Physics. Reproduced with permission.^[^ ^61^ ^]^ Copyright 2010, Elsevier. Reproduced with permission.^[^ ^62^ ^]^ Copyright 2017, The National Academy of Sciences of The United States of America. Reproduced with permission.^[^ ^63^ ^]^ Copyright 2019, The National Academy of Sciences USA. The scale bars in (a, b, c, d, and e) represent 100, 50, 100, 200, and 100 µm, respectively. The white and black scale bars in (i) represent 100 µm. The deep blue MIBs denote the outer droplets of DEs, and the black ones are inner droplets of DEs. The red ones represent the SDs.

We utilized the Deformable DETR to identify microfluidic droplets in both single images and movies. For microfluidic droplet identification in a single image, quantity, and geometric statistical information of the identified microfluidic droplets can be extracted. Focused microfluidic droplet generation frequency data can be obtained through microfluidic droplets in movies. **Figure** **3** presents machine identification on microfluidic droplets in single images from various sources. The DE droplets image in Figure 3a is originally from Mettler's work.^[^ ^56^ ^]^ As shown in Figure 3a, there is a total of 23 DE droplets identified by the Deformable DETR. However, there are only 16 identified droplets marked by yellow “√” that are sent to be statistically analyzed. It can be seen the remaining identified DE droplets reside on the edge of the image with some parts missing. That is the reason why these incomplete DE droplets, though identified, failed to be sent for further analysis. In operation, we utilized length *l* (the longer side of the MIB) and width *w* of the MIB to judge whether a microfluidic droplet was complete. If length/width ratio *l*/*w* ≥ 0.8, the machine regards it as a complete microfluidic droplet and will send it for further analysis. A DE droplet is considered complete only when its outer droplet and inner droplet(s) meet the aforementioned conditions. Regarding the droplet denoted with a blue “×” in Figure 3a, it eludes detection by the machine despite presenting itself as an intact entity upon visual human inspection. Similarly, in the case of the droplet highlighted by a red dotted circle in Figure 3b, the algorithm fails to recognize the outer droplet component, even as it successfully identifies the inner droplet. Consequently, the data associated with these droplets remain unaccounted for in subsequent statistical analysis due to either total nondetection or partial misidentification.

In the DE statistical analysis, outer and inner droplet diameters are calculated based on the MIBs. For a DE containing only one inner droplet as shown in Figure 3a,b,c,d,g,h,i, its concentricity τ (calculated by Equation (4)) and core/shell ratio δ (calculated by Equation (5)) are provided.

(4) $$\tau =1-\frac{d_{i-o}}{D_{o}}$$

(5) $$\delta =D_{i}/D_{o}$$

where *d*_i‐o_ is the distance between the center points of MIBs for the inner droplet and the corresponding outer droplet. *D*_o_ is the determined diameter of the outer droplet, calculated by (*l*_o_ + *w*_o_)/2 where *l*_o_ and *w*_o_ are the length and width, respectively, of the identified MIB for the corresponding outer droplet. The diameters of all other droplets are also calculated in the same manner. Similarly, *D*_i_ is the determined diameter of the inner droplet based on the MIB for the inner one.

**Figure 4:**
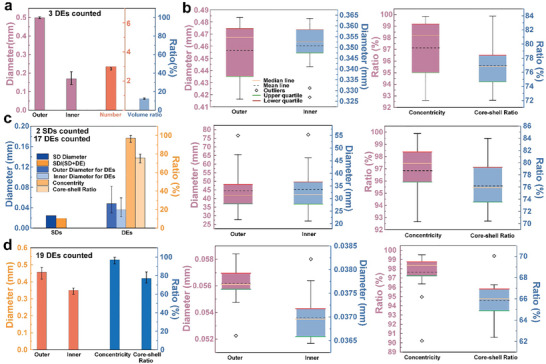
Detailed data information for microfluidic DEs in Figure 3f,g,h,i. Data distribution and box plots for microfluidic droplets in a) Figure 3f,^[^ ^60^ ^]^ b) Figure 3g,^[^ ^61^ ^]^ c) Figure 3h,^[^ ^62^ ^]^ and d) Figure 3i.^[^ ^63^ ^]^

For a DE containing more than one inner droplet as shown in Figure 3e, the previous parameters τ and δ are replaced by the inner droplet number *n* and volume ratio η calculated by $\eta =n\;\pi \;D_{i}^{3}/D_{o}^{3}$. The value of *n*, as illustrated in Figure 3e, is determined to be 3.22, with a maximum value of 4 and a minimum of 2. It indicates the proposed Deformable DETR cannot always detect all the inner droplets (see Discussion section for reasons and limitations). As illustrated in Figure 3c,d, when the sample size comprises fewer than ten droplets, the analytical output is condensed to display solely the mean value, accompanied by the maximum and minimum extremes, represented graphically through simple bar charts. Conversely, in scenarios where the dataset encompasses more than ten droplets, as depicted in Figure 3a,b,e, the data visualization expands to include additional statistical insights beyond the basic bar charts. Here, box plots^[^ ^64^ ^]^ are employed to elucidate the distributional characteristics of the droplet parameters. These box plots offer a comprehensive overview, revealing outliers, the median, mean, upper quartile, and lower quartile values, thereby providing a nuanced understanding of the statistical patterns inherent in the droplet population. Comprehensive details pertaining to each droplet's parameters are tabulated and catalogued in Tables S5 and S6 (Supporting Information). For the droplets delineated in Figure 3f,g,h,i—those encased within dotted orange boxes—the statistical data are demonstrated in **Figure** **4** as bar charts. This compilation ensures that all pertinent statistical information is accessible for further scrutiny and analysis. Intriguingly, in Figure 3i, originally from Zhang's work,^[^ ^63^ ^]^ the alphabetic label “C” was mistakenly interpreted as a DE droplet, a discrepancy that becomes apparent under magnification. From a computer vision perspective, this misclassification makes sense given the shared feature of “a circle within a circle,” which can lead to confusion between different elements. The statistical information for Figure 3i, as outlined in Table S6 (Supporting Information), has been adjusted to eliminate this misidentification through using a corrected image without the “C” as shown in Figure S11 (Supporting Information). Please refer to Note S10 (Supporting Information) for more machine identification results for a broader range of application contexts. An overall evaluation of the Deformable DETR's performance is described in Note S11 (Supporting Information). Compared with the ground truths, the average relative errors of the established Deformable DETR are within 4% and the average detection precisions are above 94%.

Besides the results from previous references, we also adopt the single‐image‐based identification technology to characterize the self‐fabricated microencapsulated PCMs and SD sizes. Please refer to Note S12 (Supporting Information) for detailed information.

#### 2.3.2 Movie‐Based Identification

**Figure 5:**
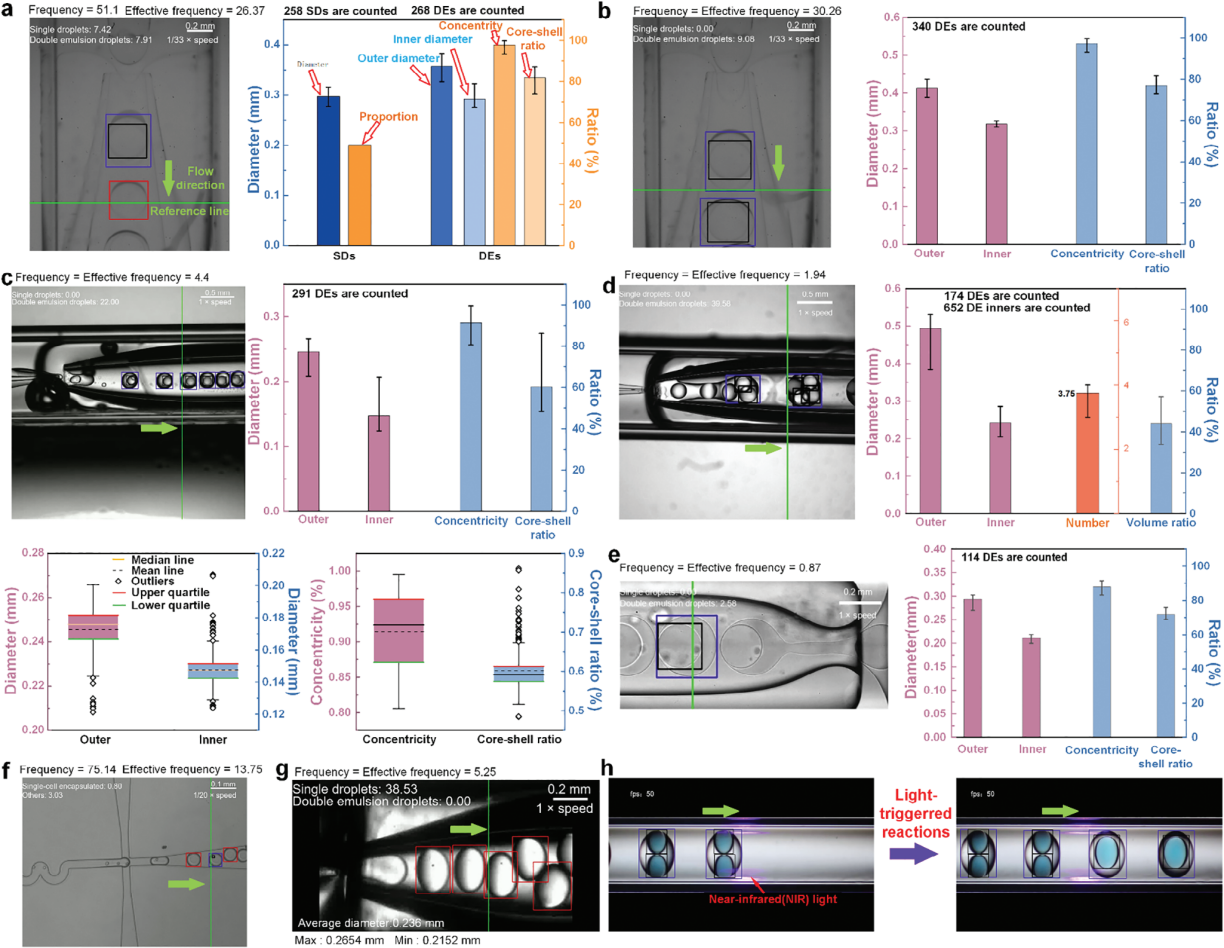
Movie‐based machine Identification of microfluidic droplets and analysis from multiple scenarios. a–d) Identification of DE and SD droplets and statistical analysis based on our own experiments. In (a), *Q*_o_, *Q*_m_, and *Q*_i_ = 166.6, 50.00, and 33.33 µL min^−1^. In (b), *Q*_o_, *Q*_m_, and *Q*_i_ = 100.0, 50.00, and 50.00 µL min^−1^. In (c), *Q*_o_, *Q*_m_, and *Q*_i_ = 100.0, 300.0, and 300.0 µL min^−1^. In (d), *Q*_o_, *Q*_m_, and *Q*_i_ = 100.0, 400.0, and 700.0 µL min^−1^. e) Identification of DE droplets and statistical analysis based on Utada et al.’s work.^[^ ^65^ ^]^ Reproduced with permission.^[^ ^65^ ^]^ Copyright 2005, American Association for the Advancement of Science. f) Identification of single cell encapsulation using our own experiments. g) Identification of SD droplets based on Kim et al.’s work.^[^ ^57^ ^]^ Reproduced with permission.^[^ ^57^ ^]^ Copyright 2014, Elsevier. h) Identification of DE droplets before and after light‐triggered reactions based on Chen et al.’s work.^[^ ^66^ ^]^ Reproduced with permission.^[^ ^66^ ^]^ Copyright 2023, Elsevier. The identification images in (a–h) are representative still images for different scenarios in Movie S2 (Supporting Information). The green line in each image functions as a reference line, which helps to calculate the generation frequency of the microfluidic droplets. The green arrow represents the flow direction of the microfluidic droplets.

Movie‐based identification results and statistics are presented in **Figure** **5**. Please refer to Note S13 (Supporting Information) for detailed operational mechanism of the movie‐based identification process. Movies in Figure 5a–d,f are from our own experiments. As shown in Figure 5a, DEs and SDs are generated alternatively. For statistical analysis of the SDs, the SD diameter and SD proportion in all generated droplets can be offered. For the DEs, diameters of the outer and inner droplets, concentricity, and core/shell ratio are statistically analyzed. An advantage of movie‐based identification, as mentioned above, is that the droplet generation frequency can be obtained. The frequency (*N*/*T*) can be calculated by monitoring the number of droplets *N* and the time *T*, it takes for them to pass over the green reference line. In our model, the droplet species traveling across the line can also be identified where the overall frequency is calculated to be 51.1 Hz (marked by the red dotted box) and the effective frequency (DE generation frequency) is 26.4 Hz (marked by the blue dotted box). Different from Figure 5a,b shows the condition where DEs are produced continuously, only parameters of DEs are statistically offered as shown in the right‐hand side of Figure 5b. For the scenario involving continuously generated droplet electrodes (DEs), the overall frequency is equivalent to the effective frequency, which is measured at 30.3 Hz.

The identifications of liposomal DEs are presented in Figure 5c,d. As shown in Figure 5c, DEs with a single core are detected with a frequency equal to the effective frequency of 4.4 Hz, indicating a 100% manufacturing success rate for liposomal DEs. Liposomal DE identifications with multiple cores (4 cores) are demonstrated in Figure 5d, also showing a 100% manufacturing success rate. The outer diameters of the droplets with multiple cores (≈0.50 mm) are more than 100% larger than those with a single core (≈0.24 mm). The average identified inner droplet number in Figure 5d is 3.75, which is similar to the phenomenon in Figure 3e. Although the nanoberry‐laden inner droplets diameters (in Figure 5c) are generally much smaller than those in DEs with multicores (in Figure 5d), the distinction in the identified diameters of the inner droplets in Figure 5c is particularly large (from 0.123 to 0.208 mm). We use box plots to further present the statistically distribution of the four parameters (outer and inner droplets diameter, concentricity, and core/shell ratio). We can see many outliers in the plots. For the movie‐based identification, the same target will be identified multiple times so that the rhombus‐marked outliers only come from limited identified targets. Here, the detected abnormally large inner droplet is shown in Figure S17 of Note S14 (Supporting Information), which, obviously, comes from an identification error (see Discussion section for reasons and limitations). Figure 5f shows a representative still image for identification of single cell encapsulated droplets in our experiments. The frequency of generated droplets there is calculated to be 75.1, while the effective frequency, the frequency of produced droplet encapsulated with a single cell, is ≈13.8. It indicates the single cell encapsulation rate (success rate) is 18.4%.

Figure 5e presents the identification results from a video sourced from the work of Utada et al.^[^ ^65^ ^]^ The statistics indicate that the identification results remain consistently stable throughout the entire video, which can be attributed to the clarity of the DEs within the video. SDs in the movie represented by Figure 5g, sourced from Kim et al.’s work,^[^ ^57^ ^]^ can also be readily identified, with the frequency measured at 5.3 Hz and the average diameter at 0.236 mm, along with maximum and minimum diameters of 0.265 and 0.215 mm, respectively. Figure 5h demonstrates the identification in a movie sourced from the work of Chen et al.,^[^ ^66^ ^]^ where dynamic microreactions within DEs can be detected. The reaction is initiated by exposure to near‐infrared (NIR) light. Prior to exposure to the NIR light, the DEs exhibit multiple cores. Subsequently, these multicore structures transform into single‐core configurations. The machine is capable of identifying the DEs both before and after exposure to the NIR light, illustrating its potential for monitoring microfluidic droplet‐based reactions. For more machine identification in extended scenarios^[^ ^63^, ^66^, ^67^, ^68^ ^]^ please refer to Movie S3 (Supporting Information).

#### 2.3.3 Web‐Based MDIA and Transfer Learning

**Figure 6:**
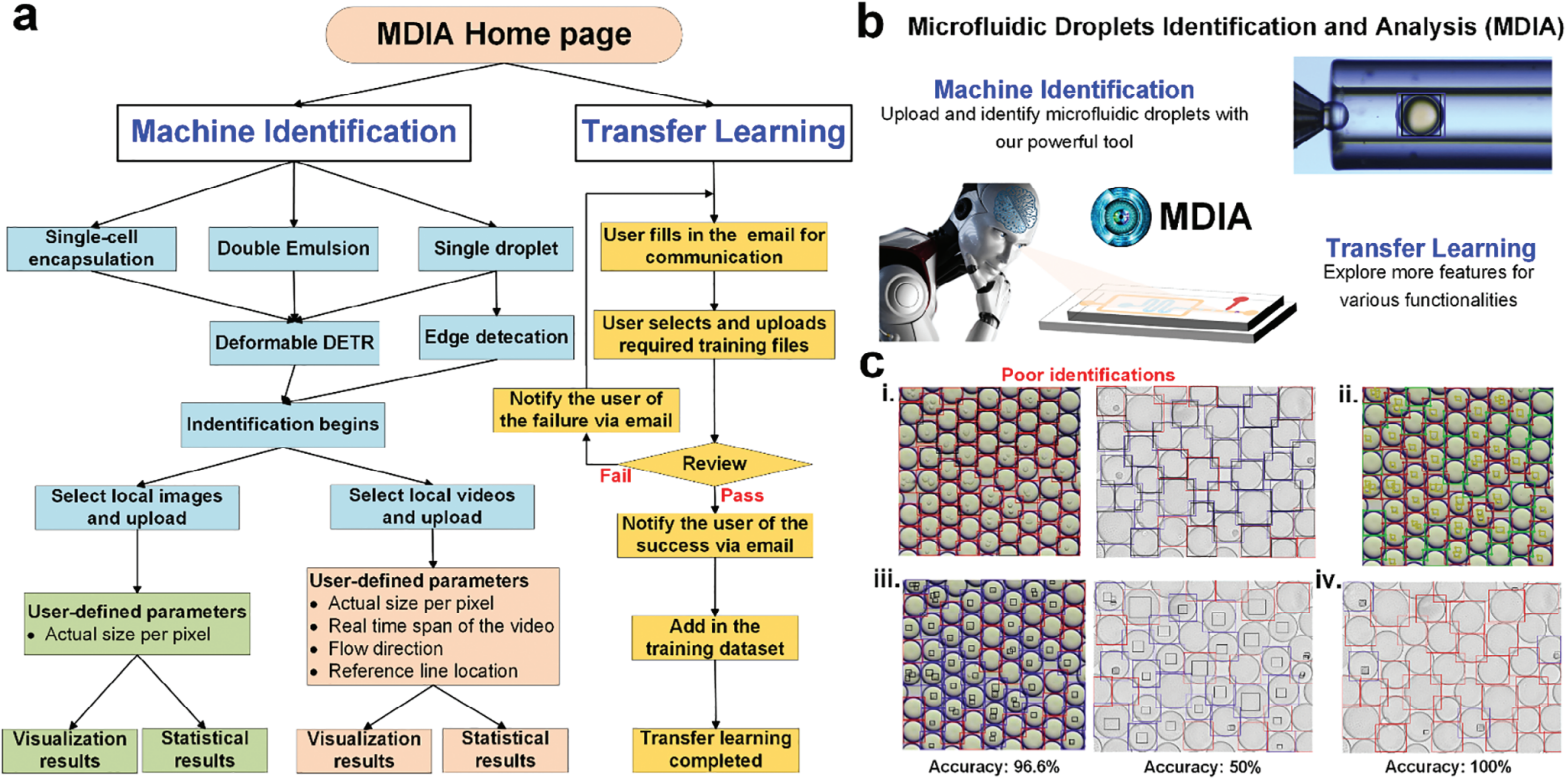
Web‐based MDIA and transfer learning. a) Flowchart of the Web‐based MDIA software development. b) Homepage of the Web‐based MDIA. c) Transfer learning processes and guidelines. i) Poor identification results without transfer learning. The left‐hand image is from Wang's work^[^ ^33^ ^]^ and the right‐hand one is from our experiments. ii) Demonstration of manually labeled image originally from Wang's work (resolution: 282 × 264 pixels).^[^ ^33^ ^]^ iii) Identification results after transfer learning using the original image^[^ ^33^ ^]^ with human annotation. iv) Improved identification results with transfer learning using Wang's image^[^ ^33^ ^]^ with enhanced resolution (resolution: 564 × 529 pixels). Reproduced with permission.^[^ ^33^ ^]^ Copyright 2021, American Association for the Advancement of Science. The task in (c) is to identify the single cell encapsulated droplets, where the sizes of these droplets are not of interest. Besides, scale bars within the images may be disruptive to identification processes. Therefore, scale bars are not provided in (c).

Based on the detection and statistical algorithms described above, we have developed an open‐source, web‐based software tool, MDIA (http://mdia.nat300.top), for the identification of microfluidic droplets. This tool is designed to address the needs of the widest possible community of researchers and industry professionals engaged in work involving microfluidic droplets. **Figure** **6**a shows the flowchart of MDIA's operation mechanism and Figure 6b presents the home page of the MDIA. There are two main functions: 1) machine identification, and 2) transfer learning. In the machine identification, the user can select a proper module among single cell encapsulation, double emulsion, and single droplet. Deformable DETR can be applied to these three modules and the algorithm of edge detection can also be selected as a main identification algorithm when Deformable DETR fails to identify single droplets (see Note S17 (Supporting Information); and Discussion). After selecting the desired identification algorithm, the identification process begins with selecting and uploading local images or videos containing the microfluidic droplets to be identified. When uploading an image, the actual size per pixel needs to be input as a user‐defined parameter for further statistical analysis. When uploading a movie, besides the actual size per pixel, three other user‐defined parameters (real‐time span of the video, flow direction, and reference line location) need to be input. Finally, visualization and statistical results are generated. Movie S4 (Supporting Information) illustrates the guidance and operational procedures of the MDIA for droplets identification and analysis.

Despite our training dataset encompassing a wide range of morphological features of microfluidic droplets, there may still be cases of suboptimal identification under specific circumstances. Therefore, the MDIA incorporates a transfer learning functionality. Users can annotate their own droplets to upload and add to our training dataset, thereby improving their own identification results. The flowchart of the transfer learning functionality is also shown in Figure 6a. The supplemental training materials from users need to be reviewed and approved by us. Figure 6c illustrates the necessity of implementing the transfer learning functionality. As our previous training dataset did not include the clustering state of single cell encapsulated droplets alongside many other droplets without cell encapsulation or with multiple cells encapsulated, the identification results from Wang's work^[^ ^33^ ^]^ and our experiments, as shown in Figure 6c(i), are unsatisfactory. Figure 6c(ii) presents the original image from Wang's work (resolution: 282 × 264 pixels)^[^ ^33^ ^]^ with manual annotations (with a minimum resolution of 5 × 5 pixels for the manually annotated objects), where single cell encapsulated droplets alongside various other types of droplets are classified appropriately. Shown in Figure 6c(iii), the identification results for the same annotated image (left‐hand side) presents a 96.6% accuracy (see Note S11 for the calculation of accuracy, Supporting Information). However, the identification for the image from our experiment (right‐hand side) is still unsatisfactory with the identification of only 50%. That means satisfactory identification only occurs in training datasets, manifesting a limited universality. We suspect the much higher resolution (1591 × 1251 pixels) of our experimental image causes this situation. We enhanced the resolution of Wang's image to 564 × 529 pixels (see Note S15 for the image resolution enhancement method, Supporting Information) and then did manual annotations again (with a minimum resolution of 11 × 13 pixels for the manually annotated objects). We updated our training dataset to become the initial state and added the manual annotated high‐resolution image for training again. Note S16 (Supporting Information) shows improved identification results with a 100% accuracy for the image from our experiment. Figure 6c(iv) shows that the identification accuracies for Wang's images^[^ ^33^ ^]^ (both low‐ and high‐resolution images) are also 100%, which is a significant improvement compared to the previous results (Figure 6c(i)). Therefore, to successfully apply transfer learning in this scenario, it is crucial to adapt the existing model to handle new data by updating the training dataset with manually‐annotated high‐resolution images (with the minimum 140 pixels for manually‐annotated target). Movie S5 (Supporting Information) details the guidance and procedure to conduct transfer learning in MDIA.

## 3 Discussion

The Deformable DETR is able to identify microfluidic droplets in multiple scenarios (from real applications to numerical simulations) for droplet morphology characterization, label‐free classification, effective target identification, frequency determination, effective droplets tracking, and microreaction monitoring. Besides DEs, general core–shell structures^[^ ^54^ ^]^ can also be detected. However, Microfluidic droplets identification from the proposed Deformable DETR may not always be accurate. There are several inherent drawbacks. First, the total identified number of microfluidic droplet targets can only be limited to 150 (default value) here. Increasing this value will result in a significant increase in both the training and identification times. Second, as illustrated in Figure S19a of Note S17 (Supporting Information), the algorithm is unable to recognize all internal droplets within multiple‐core DEs. This limitation shares similarities with the errors observed in Figure S17 (Supporting Information) and stems from the inherent limitations of digital camera imaging. Specifically, the failure to identify targets in Figure S19a (Supporting Information) and the misidentification in Figure S17 (Supporting Information) are attributed to issues related to objects being out of the camera's focal range. Therefore, possible solutions to this limitation lies in camera imaging (i.e., multiview imaging and 3D imaging techniques) and image postprocessing (i.e., image resolution enhancement techniques such as adaptive histogram equalization, Gamma correction, Gaussian filtering, bilateral filtering, etc.) technologies. Third, the proposed Deformable DETR may erroneously classify SDs as DEs, as illustrated in Figure S19b(i) of Note S17 (Supporting Information). Therefore, the basic edge detection, validated in Figure S19b(ii) of Note S17 (Supporting Information), can be alternatively selected as another identification algorithm to recognize SDs in the MDIA.

Given the vast variability in the morphology of microfluidic droplets, we must define the scope of applicability for our work. Specifically, the established MDIA is applicable to detect and analyze microfluidic SDs and DEs as well as cell‐encapsulated microfluidic droplets.

## 4 Conclusion

In summary, we have developed a robust machine vision methodology for the identification and analysis of microfluidic single and double emulsion droplets. Leveraging the Deformable DETR algorithm, our approach provides accurate detection of microfluidic droplets and general core–shell structures in diverse scenarios with detection relative error less than 4% and detection precision larger than 94%. The MDIA web‐based tool we have created supports transfer learning, allowing users to customize the droplet identification process to their specific needs. The capability, scalability, and universality of MDIA will be expanded for broader use as more training data is input from users worldwide. With its ability to quickly quantify droplet characteristics, such as diameter, number, frequency, concentricity, core/shell ratio, MDIA offers a valuable resource for advancing digital microfluidic research and applications. The open‐source MDIA has great potential to be utilized as a powerful in situ real‐time tool, monitoring, classifying, and actively controlling the generated microfluidic droplets for synthesizing, label‐free sorting, tracing, automatic statistical analysis, and automated microfluidics experimentation and manufacture.

## 5 Experimental Section

### Model Training and Computer Configuration

After the parameter optimization process described in Note S1 (Supporting Information) (epoch = 80 and learning rate = 0.003), the model was trained via a custom‐built image inventory comprised of 521 (including the one from the transfer learning section in this paper) manually labeled microfluidic droplets images acquired from own experiments and other extensive publications. The process of manually labeling images for model training, known as image annotation, was carried out by a team of trained annotators over a period of 3 months using an open‐source annotation software (LabelMe, MIT, USA). The algorithm was established on a computer with a Intel(R) Core(TM) i9‐10850K CPU @ 3.60 GHz, a GPU of NVIDIA GeForce RTX 3070, and a RAM of 32 GB DDR4.

### Fabrication Process of the Flow‐Focusing Microfluidic Chip for Producing o‐w‐o DEs

The microfluidic chip consists of three main components: an inner‐phase capillary, a collection capillary, and an outer square tube. Both of the capillaries are made of high‐quality borosilicate glass with an outer diameter of 1.0 ± 0.1 mm and an inner diameter of 0.75 ± 0.1 mm. The square tube has an internal dimension of 1.1 ± 0.1 mm and an external dimension of 1.3 ± 0.1 mm. First a Narishige PC‐100 puller was used to taper the tips of both capillaries to 1 µm. Subsequently, the tips of the inner‐phase and collection capillaries were polished using Narishige MF‐2 polisher to ≈300 ± 10 and 400 ± 10 µm, respectively. The two capillaries were then inserted into the square tube. It is crucial to ensure that the tip of the inner‐phase capillary aligns with the tip of the collection one, with a separation distance of ≈300 ± 10 µm. The two capillaries must be coaxially aligned within the square tube, meaning they are centered. After securing the positions of the three glass tubes, one end of the inner‐phase capillary with a 23‐gauge needle (having an inner diameter of 0.33 mm and an outer diameter of 0.63 mm) was sealed to serve as the inlet for the inner phase liquid. The upper end of the square tube with a 23‐gauge needle to act as the inlet for the middle phase fluid was also sealed, and the lower end of the square tube was also sealed with a 23‐gauge needle to function as the inlet for the outer phase.

### Fabrication Process of the Flow‐Focusing Microfluidic Chip for Producing Liposomal DEs

The overall structure of the flow‐focusing microfluidic chip used in this study is similar to that employed for the production of o‐w‐o DEs. However, the dimensions differ slightly. Specifically, the square tube used in this setup has an internal dimension of 1.0 ± 0.1 mm and an external dimension of 1.1 ± 0.1 mm. To produce different types of double emulsions (single‐core DEs and multicore DEs), three sizes of tapered inner‐phase tips were utilized: 100 ± 10, 200 ± 10, and 300 ± 10 µm. The tip of the collection capillary was also maintained at 300 ± 10 µm. The separation distance between the inner‐phase and collection capillaries was set to match the size of the utilized tapered inner‐phase tip.

### Fabrication Process of the Microfluidic Coextrusion Device

The device was fabricated using two borosilicate glass capillaries of different diameters and two corresponding dispensing needles mounted on a glass surface. To enhance interface stability and prevent unwanted rupture of the double emulsion droplets over time, the inner capillary (with an outer diameter of 0.58 ± 0.05 mm and an inner diameter of 1.00 ± 0.1 mm) was treated to become hydrophobic by immersing it in a 5% octadecyl trichlorosilane acetylacetone solution for 8 h. This treatment was appropriate given that the utilized PCM paraffin is an alkane,^[^ ^69^, ^70^ ^]^ which exhibits hydrophobic properties. Similarly, the outer capillary (with an outer diameter of 1.20 ± 0.1 mm and an inner diameter of 1.00 ± 0.1 mm) was treated to become hydrophilic by immersing it in a 5% sodium hydroxide absolute ethanol solution for 8 h. The two borosilicate glass capillaries were then concentrically nested and secured on the glass slide. The inlets of the capillaries were bonded to the dispensing needles (23‐gauge needles) using a transparent epoxy resin, with a preformed groove to seal the joint and prevent leakage.

### Fabrication Process of the Flow‐Focusing Microfluidic System for Generating Single Cell Encapsulated Droplets

The microfluidic chip design was created using CAD software, and the blueprint was sent to a specialized company for mask fabrication. In the photolithography process, a silicon wafer was coated with SU‐8 resist via spin coating at 500 rpm for 30 s and 2750 rpm for 60 s, creating a uniform ≈30 µm coating. The coated wafer underwent a soft bake at 65 °C for 1 min and 95 °C for 4.5 min, followed by UV exposure at 9.8 mJ cm^−^^2^ for 15.6 s. Postexposure baking was conducted at 65 °C for 1 min and 95 °C for 4.5 min. The wafer was then developed in SU‐8 developer for 4.5 min, rinsed with isopropanol and deionized water, and hard baked at 150 °C for 5 min. Before soft lithography, the patterned wafer was treated with 20 µL of FOTS in a desiccator overnight to create an antisticking layer. A 10:1 weight ratio of PDMS monomer to initiator was mixed to a total weight of 35 g, poured onto the wafer, degassed, and cured at 85 °C overnight. The cured PDMS was peeled off, cut, and punched with inlets, outlets, and electrodes. The PDMS slab was bonded to a glass slide using plasma activation for 1 min, followed by firm bonding at 85 °C for 1 h. The microfluidic channels were then treated with Aquapel and purged with compressed air to remove excess reagent.

### Statistical Analysis

The data of the detected microfluidic droplets were presented using the method of box plots. The sample size was contained in each data presentation figure. For example, the 16 DEs counted in Figure 3a means the sample size there is 16.

## Conflict of Interest

The authors declare no conflict of interest.

## Author Contributions

J.‐X.W., H.W., and H.L. contributed equally to this work. S.Y., M.Y., and J.‐X.W. conceived the project. J.‐X.W., H.L., and W.Y. performed the microfluidic synthetic methodology and experiments. J.‐X.W., H.W., Y.M., and W.Y. wrote python programs and developed/improved the software MDIA. J.‐X.W., F.X.L., and B.C. analyzed the experimental data and data from other publications. S.Y. and M.Y. contributed supervision and project management. All the authors discussed the results and co‐wrote the manuscript.

## Supporting information



Supporting Information

Supplemental Movie 1

Supplemental Movie 2

Supplemental Movie 3

Supplemental Movie 4

Supplemental Movie 5

## Data Availability

The data that support the findings of this study are available from the corresponding author upon reasonable request.
