# Supplementary material for: A Machine Vision Perspective on Droplet‐Based Microfluidics
Source: Adv Sci (Weinh). 2025 Jan 1;12(8):2413146. doi: 10.1002/advs.202413146 (PMC11848540; doi:10.1002/advs.202413146)
Supplement: Supplementary file 1 — Supporting Information [file ADVS-12-2413146-s004.docx]

Supporting Information

A Machine Vision Perspective on Droplet-Based Microfluidics

Ji-Xiang Wang, Hongmei Wang, Huang Lai, Frank X. Liu, Binbin Cui, Wei Yu, Yufeng Mao, Mo Yang,* Shuhuai Yao*

**This file includes:**

Supplementary Notes 1-17

Figures S1-S19

Tables S1-S7

References

Supplementary Movies 1-5

**Supplementary Notes**

**Note 1**

**Detailed Mechanisms of Deformable DETR**

| 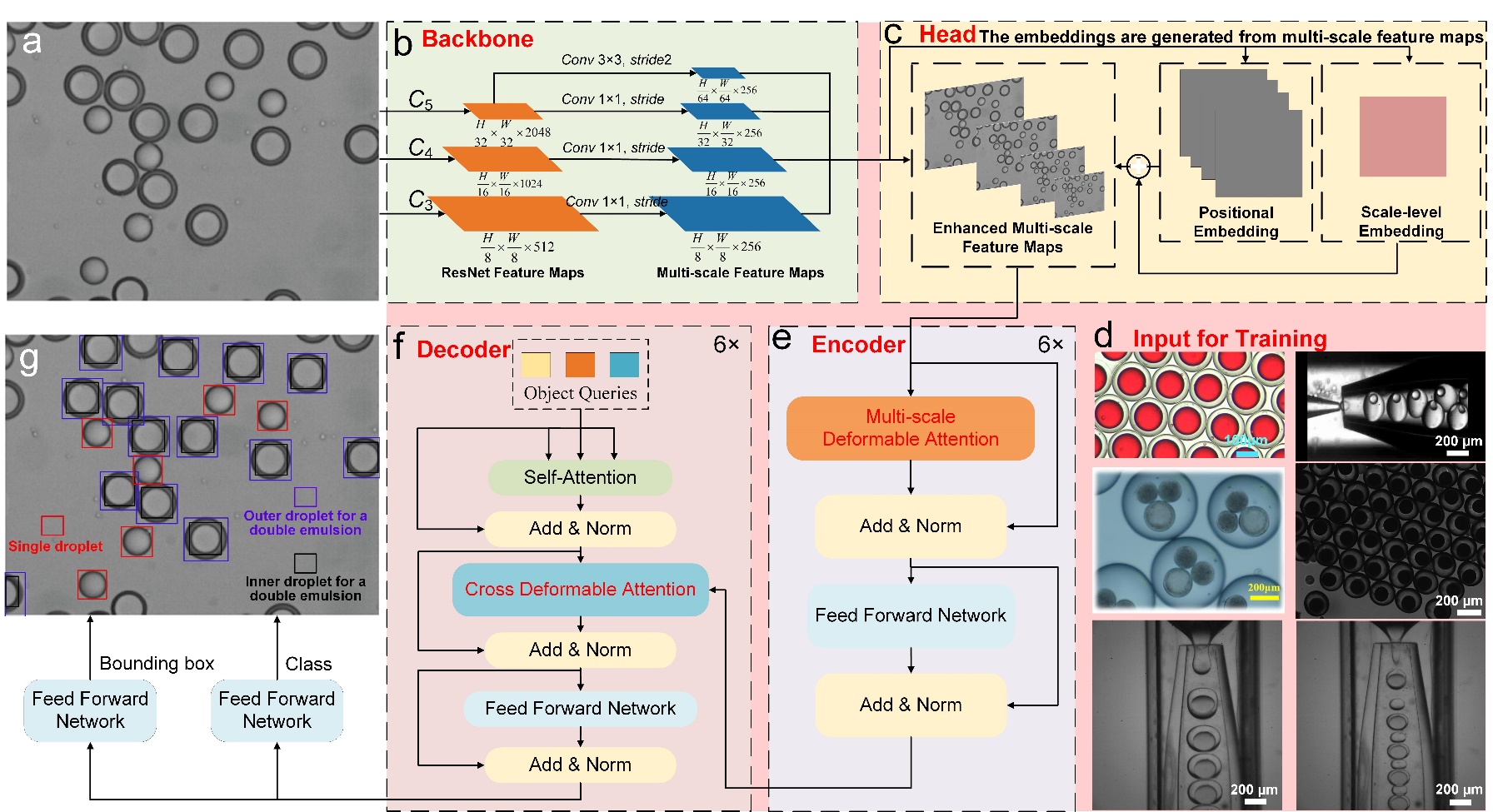  **Fig. S1. The workflow of the established Deformable DETR to identify microfluidic droplets.** It ingeniously integrates four core components: **b** Backbone, **c** Head, **e** Encoder, and **f** Decoder, facilitating advanced microfluidic droplets detection in images. This architecture meticulously engineers a pipeline that begins with feature extraction, progresses through sophisticated encoding, and culminates in precise object localization and classification. Before conducting the microfluidic droplet identification, the Deformable DETR training with microfluidic droplet morphology knowledge (typically shown in **d**) under multiple scenarios is required. With the Deformable DETR, the microfluidic droplets in **a** original image can be identified and classified, demonstrated in **g**. |
| --- |

The detailed operational mechanism of the utilized Deformable DETR is described as follows. In the process of identifying a droplet image with dimensions of width *W* pixels and height *H* pixels, featuring three color channels (RGB), recognition commences with the Backbone, primarily aimed at extracting MSFMs $\mathbf{\{x}^{l}\mathbf{\}}$(*l* =1, 2, and 3) from the outputs of its *C*_3_, *C*_4_, and *C*_5_ layers in ResNet^1^ (obtained via a 1 × 1 stride 1 convolution). These layers embody feature representations at varying depths within the network, with *C*_5_ furnishing the highest level of abstracted features. Besides, the fourth feature map with the lowest resolution $\mathbf{x}^{4}$ is acquired through a 3 × 3 stride 2 convolution on the *C*_5_ layer. The four resulting feature maps all have 256 channels, with their widths and heights scaled down to 1/8, 1/16, 1/32, and 1/64 of the original image's pixel dimensions, respectively, collectively forming the multi-scale image feature maps (MSFMs) $\mathbf{\{x}^{l}\mathbf{\}}$(*l* =1, 2, 3, and 4). The MSFMs are capable of capturing image details across various scales, enhancing the ability to handle microfluidic droplets of diverse sizes and shapes, thereby improving recognition and localization accuracy^2^.

The maps are then transmitted to the Head to construct positional embeddings and scale-level embeddings, through which we obtain enhanced MSFMs (EMSFMs) $\mathbf{\{x}_{^{embed}}^{l}\mathbf{\}}$ (*l* =1, 2, 3, and 4), defined by $\mathbf{x}_{embed}^{l}=\mathbf{x}^{l}+\text{pos\_embed(}\mathbf{x}^{l}\text{)}+\text{scale\_embed(}\mathbf{x}^{l}\text{)}$.^3^ By incorporating these embeddings, $\mathbf{\{x}_{^{embed}}^{l}\mathbf{\}}$ enhance the machine-vision’s accuracy and robustness in identifying microfluidic droplets, enabling more efficient recognition and localization across diverse microfluidic scenarios.

$\mathbf{\{x}_{^{embed}}^{l}\mathbf{\}}$ form the input of the subsequent six-layer Encoder (marked by 6× in Fig. S1e) where global context from the diverse scale representations can be captured. In each layer, the multi-scale deformable attention module (MSDAM) processes the input of $\mathbf{\{x}_{^{embed}}^{l}\mathbf{\}}$ as^3^:

| , | (S1) |
| --- | --- |

where $\mathbf{z}$ is the feature vector obtained by concatenation of the four EMSFMs, as denoted by $\mathbf{z}=\text{Concat}\left(\left\{\mathbf{x}_{\text{embed}}^{l}\right\}\right)$^3^, $\hat{p}$ is one reference point of each pixel in each EMSFM of $\mathbf{z}$, means *k*th (*k* = 1 ~ 4) sampling point at the *l*th scale in the *m*th (m = 1 ~ 8) attention head, and are the weight matrixes at *m*th attention head, and represents the attention weight. These weights are tuned through the training process, responsible for precise focus on the edges of the microfluidic droplets. All reference points in $\mathbf{z}$ undergo Eq. (S1) to form the output vector from MSDAM, which is, subsequently, normalized by a residual structure in the Add and Norm module (ANM) to mitigate gradient vanishing and explosion issues, thereby accelerating model convergence. Afterwards, the output is delivered to the feed forward network (FFN), followed by the ANM. The FFN enables the model to better capture the intricate relationships in microfluidic droplet features and enhance its learning capacity for characterizing microfluidic droplets. Then, the output enters the second layer of Encoder, repeating the above process from MSDAM to ANM.

After being processed by all six layers, the final Encoder output vector $\mathbf{x}_{encode}$ combined with object queries (denoted by $\mathbf{z}_{q}$) is sent to the Decoder. Here, $\mathbf{z}_{q}$, in the first layer of Decoder, constitutes *q* = 150 initial feature vectors that embody the model's prior assumptions about potential objects present in the image, which means the adopted machine vision can only identify a maximum of 150 microfluidic droplets in a single image. These query vectors are learned parameters refined over the training course to better align with actual objects in images. $\mathbf{z}_{q}$ goes through a self-attention module^4^ and a ANM, where $\mathbf{z}_{q,SA}$ is obtained, facilitating information exchange, enhancing global context understanding, and enabling refined feature representation for more accurate droplet classification, localization and recognition. Subsequently, $\mathbf{z}_{q,SA}$ and $\mathbf{x}_{encode}$interact with each other in a cross- deformable attention mechanism, expressed as^3^:

| , | (S2) |
| --- | --- |

where $\hat{p}_{q}$ is the reference point of the feature vector at *q*th query, is *k*th (*k* = 1 ~ 4) sampling point at the *l*th scale in the *m*th (*m* = 1 ~ 8) attention head under *q*th query. , , and are learned weights. Interacting with $\mathbf{z}_{q,SA}$, pixels related to droplets within $\mathbf{x}_{encode}$ are extracted to form $\mathbf{z}_{q,SA+CA}$, which contains more droplets information. After being processed by ANM, FFN, and ANM, as shown in Fig. S1f, the output is delivered into the second layer of Decoder as updated $\mathbf{z}_{q}$, repeating the above process through six layers and ultimately producing feature output $\mathbf{x}_{decode}$. Last, the output is transferred into two separate FFNs, with one FFN tasked to convert the information into positions (i.e., bounding boxes), and the other FFN responsible for transforming it into class labels.

**Note 2**

**Deformable DETR parameters tuning process, results, and optimization**

Initially, we adopted 520 manually labelled microfluidic droplet images as the dataset. The dataset and its description can be found in our GitHub project at <https://github.com/MicrofluidicDroplets/MDIA>. To mitigate the effects of data noise, we randomly divided all the data into training and testing datasets. The training dataset consisted of 80% (415 images) of the total data, while the testing dataset comprised 20% (104 images). Maintaining this ratio for the training set, we tuned the parameters (learning rate and epoch) of the machine vision model.

After setting the parameter each time, we trained the model with the randomly-selected manually labelled training data and utilized the trained model to identify microfluidic droplets in the remaining raw 104 images. We also compare the machine identified microfluidic droplets with manual labelled ones using an assessment system comprising three evaluation metrics (*IoU*, $\delta_{l}$, $\delta_{A}$), which have been described in Eqs. (1), (2), and (3) in the main text for quantitative comparison between each machine identified box and ground truth box (manually labelled box). Although the model has the same structure and parameters for each running time, the results typically vary primarily due to the random selection of the training data. To mitigate the fluctuations caused by this randomness, we conducted 10 runs for each parameter combination. Therefore, averaged metrics ($\overline{\overline{IoU}}$ defined by Eq. (S3), $\bar{\bar{\delta}}_{l}$ defined by Eq. (S5), and $\bar{\bar{\delta}}_{A}$ defined by Eq. (S7)) based on the 10 runs were adopted for selecting the optimal parameter combination, as listed in Table S1.

| $\overline{\overline{IoU}}=\frac{\sum_{j=1}^{10} \overline{IoU}_{j}}{10}$ | (S3) |
| --- | --- |
| $\overline{IoU}_{j}=\frac{\sum_{i=1}^{n} IoU_{i,j}}{n}$ | (S4) |

Where $\overline{IoU}_{j}$, calculated by Eq. (S4), is the average *IoU* at the *j*th (*j* = 1, 2, …, 10) run of the model. $IoU_{i,j}$ is the *IoU* value for the *i*th (*i* = 1, 2, …, *n*) microfluidic droplet at the *j*th run of the model. There is a total of *n* machine identified microfluidic droplets that can be compared with the corresponding manually labelled ones.

| $\overline{\overline{\delta}}_{l}=\frac{\sum_{j=1}^{10} \overline{\delta}_{l,j}}{10}$ | (S5) |
| --- | --- |
| $\bar{\delta}_{l,j}=\frac{\sum_{i=1}^{n} \delta_{l,i,j}}{n}$ | (S6) |
| $\bar{\bar{\delta}}_{A}=\frac{\sum_{j=1}^{10} \bar{\delta}_{A,j}}{10}$ | (S7) |
| $\bar{\delta}_{A,j}=\frac{\sum_{i=1}^{n} \delta_{A,i,j}}{n}$ | (S8) |

As shown in Table S1, the model achieves the highest *IoU* value, indicating superior overlap between predicted and ground truth segments, while maintaining the lowest values for metrics $\delta_{l}$ and $\delta_{A}$. This optimal performance was observed when the learning rate was set to 0.003 and the number of epochs was 80, suggesting a high identification accuracy.

Table S1. Detailed hyper-parameter tuning process.

| Learning Rate | Epoch | $\overline{\overline{IoU}}$ | $\bar{\bar{\delta}}_{l}$ | $\bar{\bar{\delta}}_{A}$ |
| --- | --- | --- | --- | --- |
| 0.0001 | 20 | 0.9177 | 0.0503 | 0.0545 |
| 0.0002 | 20 | 0.8844 | 0.0693 | 0.0797 |
| 0.0003 | 20 | 0.9205 | 0.0483 | 0.0468 |
| 0.0004 | 20 | 0.8188 | 0.1214 | 0.1409 |
| 0.0003 | 40 | 0.8483 | 0.1004 | 0.0661 |
| 0.0003 | 60 | 0.8824 | 0.0749 | 0.0618 |
| **0.0003** | **80** | **0.9247** | **0.0466** | **0.0417** |

**Note 3**

**Details of the establish microfluidic platforms**

| 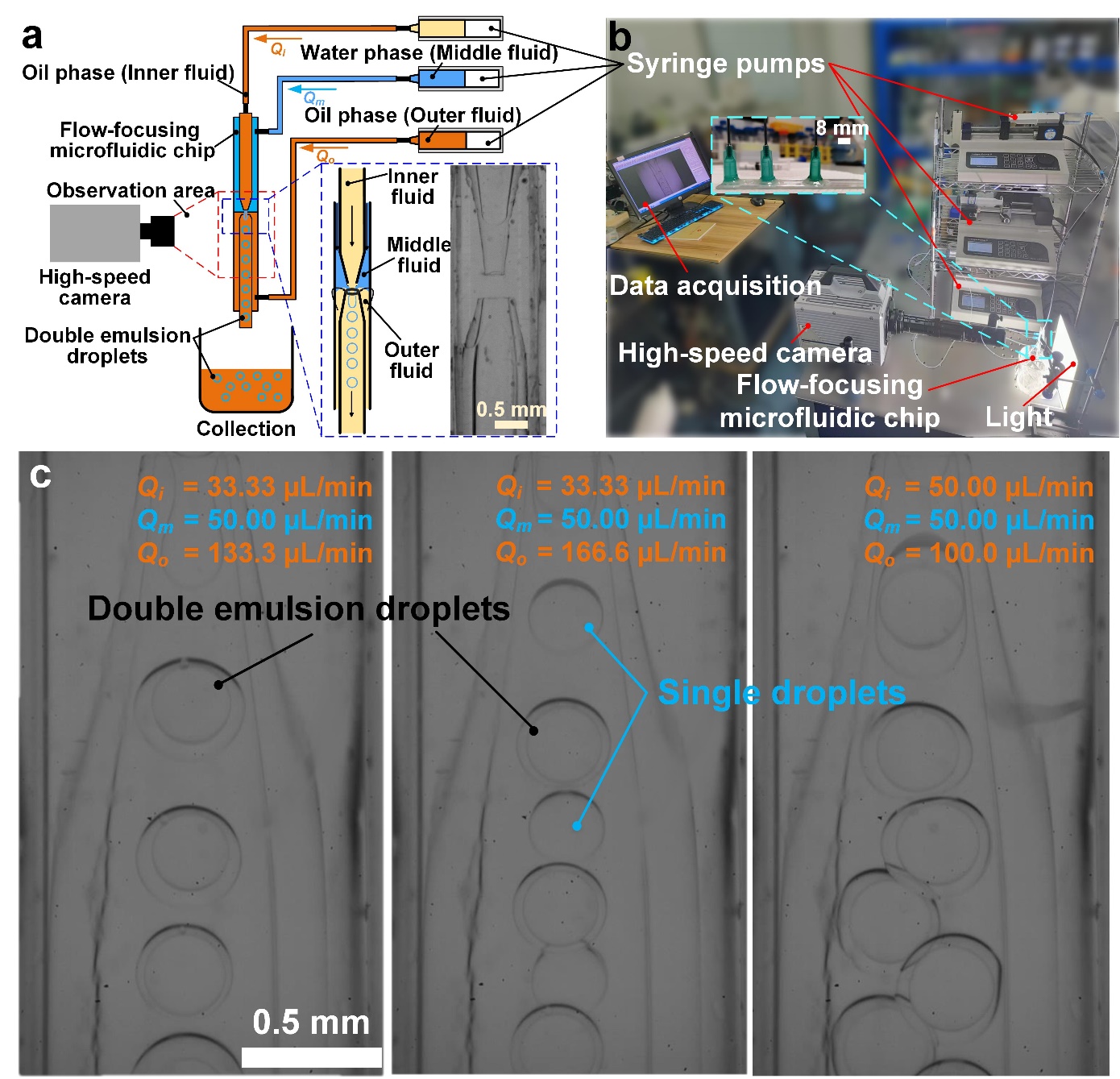  **Fig. S2. Flow-focusing microfluidic system for generating normal DE droplets.** **a** Schematic view of the overall experimental system. **b** Photographic view. The insert shows the microfluidic flow-focusing chip. **c** O-w-o DE droplets generation demonstrations in three different conditions. |
| --- |

Fig. S2 and Fig. S3 illustrates the experimental flow-focusing microfluidic systems, featuring an enlarged perspective of the employed flow-focusing microfluidic chip (See Methods for detailed fabrication process). Fig. S2 shows the generation of oil-water-oil (o-w-o) DEs, and Fig. S3 shows the production of water-oil-water (w-o-w) liposomal DEs. The microfluidic chip, demonstrated in Fig. S2a and the insert of Fig. S2b, comprises three inlets, each connected to dedicated syringe pumps designed to sequentially introduce the three-phase fluids into the microfluidic environment. To generate the o-w-o DEs, both the outer and inner phase fluids consist of silicone oil (XIAMETER PMX-200) forming the Dow Corning. Sandwiched between these phases is the middle phase, comprised of a sodium dodecyl sulfate (SDS)-water solution with the SDS concentration of 3.34% by weight (wt%). Please refer to Table S2 of Supplementary Note 4 for detailed properties of the utilized three phases. Three typical microfluidic droplet generation conditions are presented in Fig. S2c. When the volume rates of the inner (*Q_i_*), middle (*Q_m_*), and outer (*Q_o_*) phases are 2, 3, and 8 mL/h, respectively, the double emulsion formation can occur in a continuous manner as presented in the leftmost image of Fig. S2c. When *Q_o_* increases to 10 mL/h, both double emulsions and SDs can be generated in an intermittent pattern as shown in the middle image. When *Q_o_* decreases to 6 mL/h as shown in the rightmost image, double emulsions can also be continuously generated; however, they appear more crowded due to the reduced flow rate of the outer fluid, which hampers the efficient movement of the droplets.

Fig. S3a illustrates the generation mechanism of liposomal DEs, which encapsulates polypeptide-based nano-berries for DNA plasmid protection, responding to pH variations in the solution. At basic pH, polypeptides form nano-berries that encapsulate and safeguard the DNA. Conversely, at lower pH levels, the nano-berries disassemble, releasing the DNA to facilitate targeted cell transfection. Please see Ni and Chau’s work^5^ for fabricating the nano-berry. The characterization of the nano-berries is presented in Supplementary Note 5. A significant challenge is that once the nano-berries are internalized by the cells, they often fail to escape the endosome and are largely degraded by endosomal enzymes. To address this issue, we incorporate dipalmitoylphosphatidylcholine (DOPC) as solute dissolved in 1-octanol (Sigma Aldrich) as solvent to form 2mg/ml of lipid solution (middle phase), providing additional protection to the nano-berries and enhancing their endosomal escape. The inner phase is 2 wt% polyvinyl alcohol (PVA) (Sigma Aldrich) solution in deionized water, with the addition of 5 wt% of Pluronic F127 (Sigma Aldrich) as the surfactant, containing 10 v/v% of nano-berries. The pH of the solution is adjusted to 7.4 to retain the functionality of the nano-berries with NaOH (VWR Chemicals) under the pH meter (Beckman). The outer phase is 5wt% of PVA solution in deionized water (18.1MΩ) with the addition of 5wt% of Pluronic F127 as surfactant. Three typical DE generation conditions are presented in Fig. S3b.

| 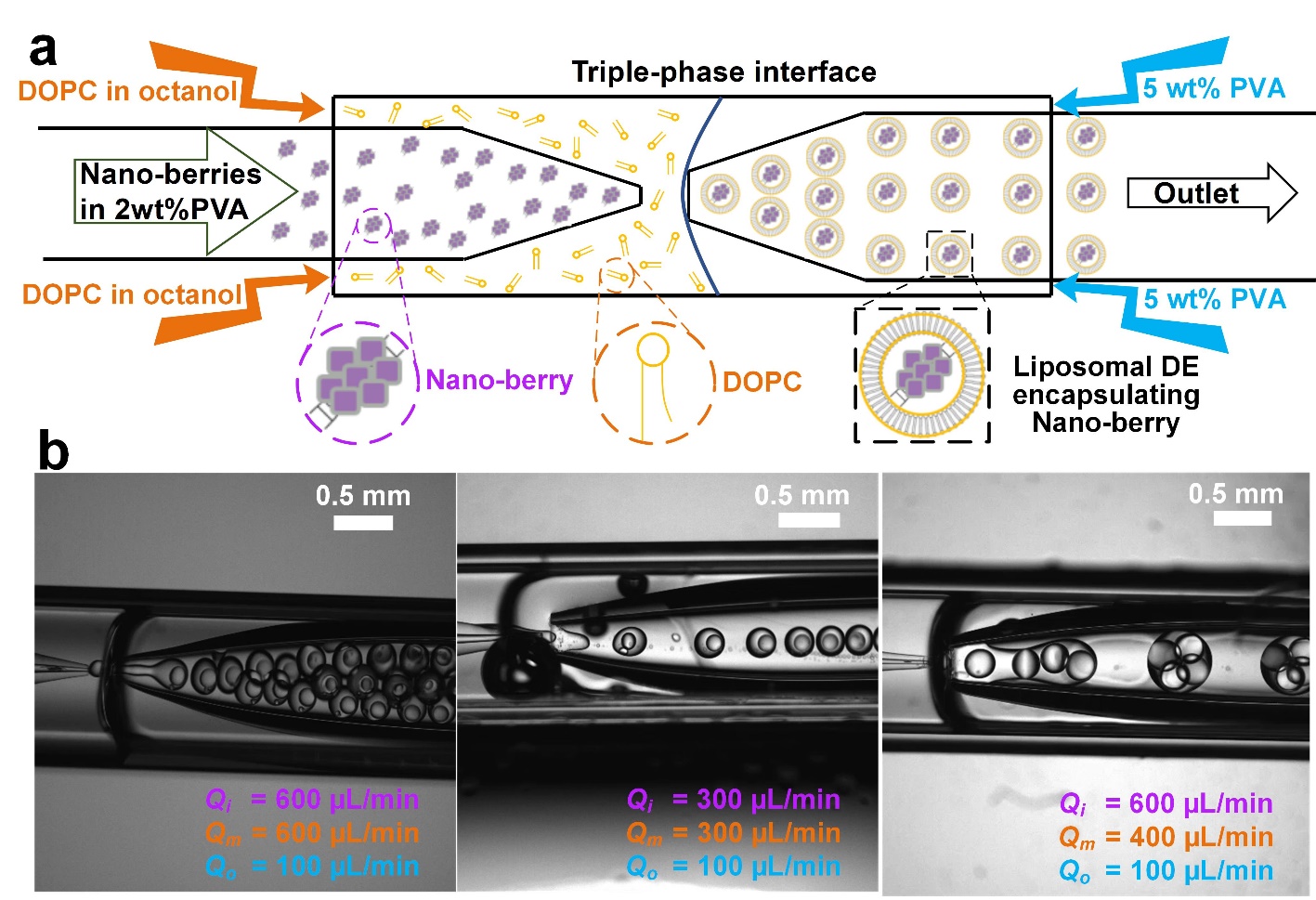  **Fig. S3. Flow-focusing microfluidic system for generating liposomal DEs.** **a** Schematics for producing liposomal DEs that encapsulates nano-berries. **b** W-o-w DE droplets generation demonstrations in three different conditions. |
| --- |

Fig. S4a and b presents photographic and schematic views of the experimental co-flowing microfluidic apparatus that produces single droplets (SDs). A syringe needle, characterized by an inner diameter (*d_i_*) of 0.09 mm and an outer diameter (*d_o_*) of 0.2 mm, was securely positioned within a square microchannel. This microchannel, defined by a hydraulic diameter of 2 mm, had its inlet connected to a syringe pump. The pump was responsible for infusing the continuous phase, comprised of silicone oil (KF-96) sourced from Shin-Etsu Chemical Co., Ltd., into the microchannel environment with measured volume rate *Q_cont_*. The syringe needle's inlet was joined to a second syringe pump. Through this pump, a precisely measured volume rate of deionized water *Q_disp_*, enriched with a specific concentration of surfactant, was dispensed as the discrete phase. The surfactant employed was sodium dodecyl sulfate SDS, identified by no. STBJ6562, and supplied by Sigma-Aldrich (Shanghai). The SDS played a critical role in modulating the interfacial tension coefficient at the boundary between the continuous and discrete phases. This interfacial tension is pivotal in the microfluidic generation of water-based micro-droplets, influencing their formation and stability. Shown in Fig. S4c, a typical SD generation process is also presented under the operation condition of *Q_cont_* = 50 mL/h and *Q_disp_* = 2.5 mL/h. Please consult Table S3 of Supplementary Note 4 for the characteristics of the silicone oil and the SDS-water solutions that were used.

| 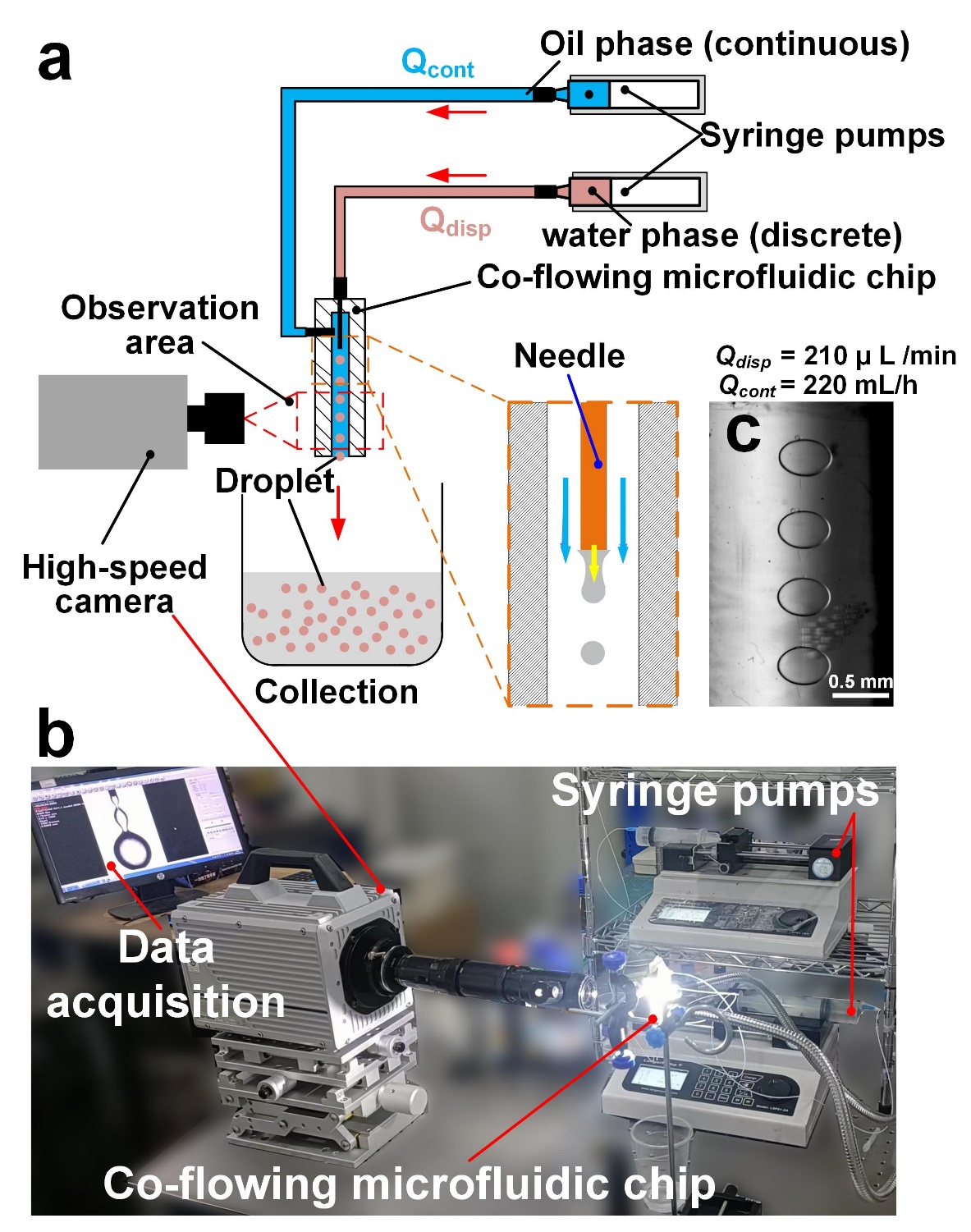  **Fig. S4. Co-flowing microfluidic system for generating SDs.** **a** Schematic view of the experimental system. **b** Photographic view. **c** SD generation demonstration at one operating condition. |
| --- |

As shown in Fig. S5a, a co-extrusion microfluidic chip was fabricated to realize phase change material (PCM) encapsulation. The chip was made of two coaxially-aligned borosilicate-based glass capillaries and two dispensing needles (See Methods for detailed fabrication process). In this scenario, a warm (40°C) liquid-phase PCM, specifically paraffin n-octadecane C_18_H_38_ (with detailed properties outlined in Supplementary Note 6) bought from Rubitherm, as the inner phase, flowed into the inner capillary of the microfluidic device. Concurrently, sodium alginate (no. S100128 from Shanghai Aladdin Bio-Chem Technology Ltd.) aqueous solution intended for the capsule shell, also in its liquid phase, was delivered into the outer capillary as the outer phase, facilitating the creation of a PCM encapsulation. During the encapsulation process, the PCM was encapsulated within a liquid aqueous solution of sodium alginate as shown in (ii) of Fig. S5b. Under the influence of gravity, the newly formed PCM capsules descended into a calcium chloride (no. C290953 from Shanghai Aladdin Bio-Chem Technology Ltd.) solution. Here, a crosslinking reaction, $\text{Ca}^{+}+\text{NaC}_{6}\text{H}_{7}\text{O}_{6}\to\text{Na}^{+}+\text{CaC}_{6}\text{H}_{7}\text{O}_{6}\downarrow$, took place facilitated by calcium cations, which interacted electrostatically with the alginate molecules. This chemical bonding resulted in the transformation of the alginate layer into a solidified calcium alginate shell, effectively enclosing the PCM within a robust capsule structure (See Supplementary Note 6 for characterization) to prevent liquid paraffin leakage.

| 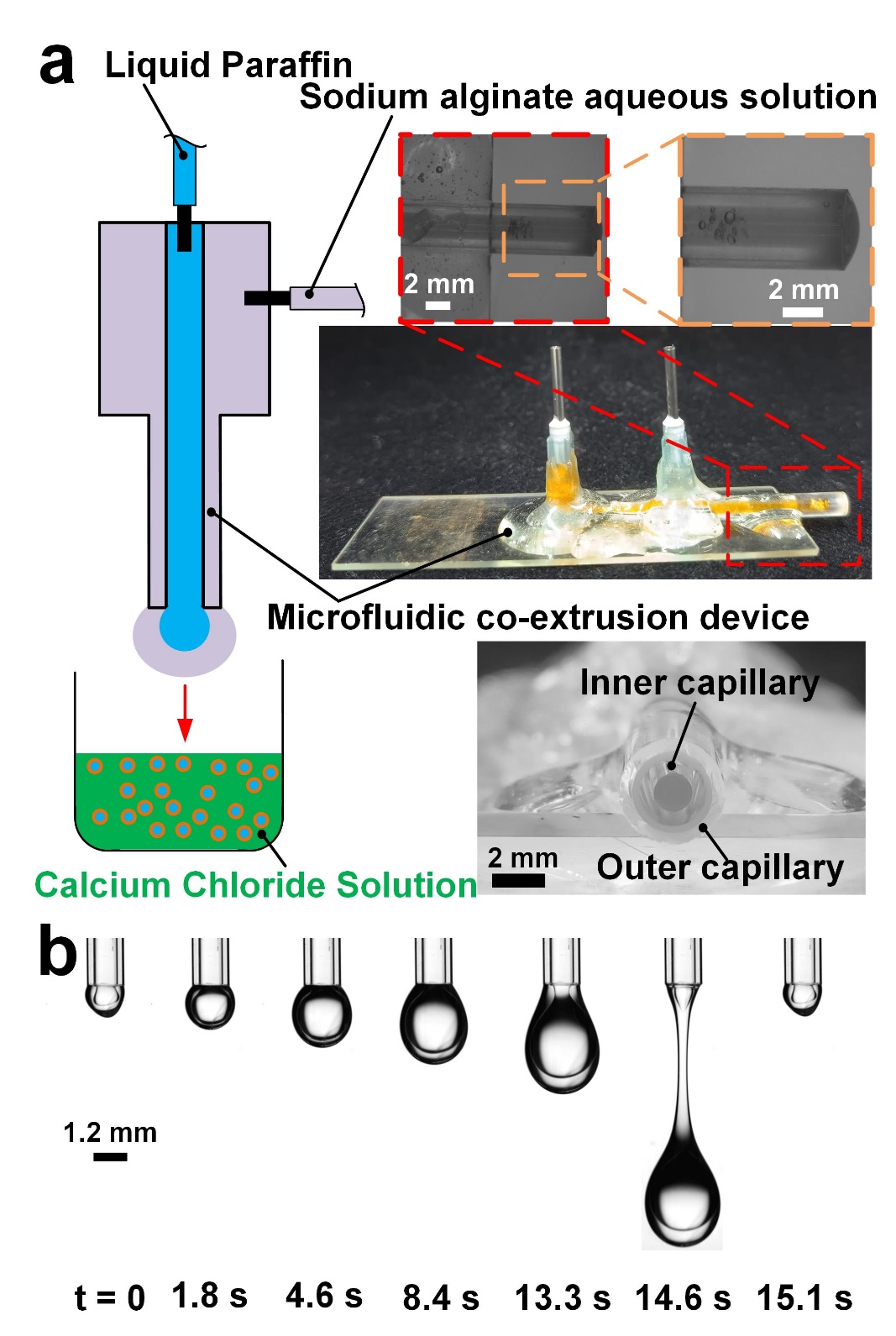  **Fig. S5. PCM encapsulations fabrication using a microfluidic co-extrusion device.** **a** Schematic and photographic views of the working mechanism of paraffin encapsulation fabrication using the co-extruding technique. Magnified view of the utilized microfluidic co-extrusion device and side view of the inner capillary inserted in the outer capillary are provided to characterize the utilized device. **b** Formation dynamics of the paraffin-sodium alginate double emulsion (*Q_o_* = 7.5 mL/h and *Q_i_* = 3.5 mL/h). |
| --- |

Fig. S6a and b illustrates microfluidic flow-focusing system for generating single-cell encapsulated droplets (See Methods for detailed design and fabrication process of the chip). As the continuous phase, a blend of high molecular weight fluorinated oil, specifically HFE 7500 from 3M Novec, combined with a commercially available surfactant known as dSurf (wt 5%) from Fluigent, was utilized. An RPMI-1640 cell culture medium from Thermo Fisher Scientific was adopted for the discrete phase. The T cell (Jurkat E6-1) was purchased from ATCC. The inlets for the continuous and discrete phases were connected to their respective fluid reservoirs. The volumetric flow rate of each phase was regulated by a dedicated pressure controller. The pressures of the continuous *P_cont_* and discrete *P_disp_* phases were set to 120 mbar. Before generating single-cell encapsulated droplets, we investigated the droplet size without using a cell suspension (See Supplementary Note 7). This preliminary study aimed to define the relationship between droplet size and pressure applied in the chip. Following this, a cell suspension at the density of 0.5 cell per droplet was prepared. The single-cell encapsulated droplet with pico-injection process was recorded under a microscope and a high-speed camera, allowing for subsequent visual observation and evaluation. Fig. S6c demonstrate a fine single-cell encapsulation dynamic process at the cross-junction of the microfluidic chip.

| 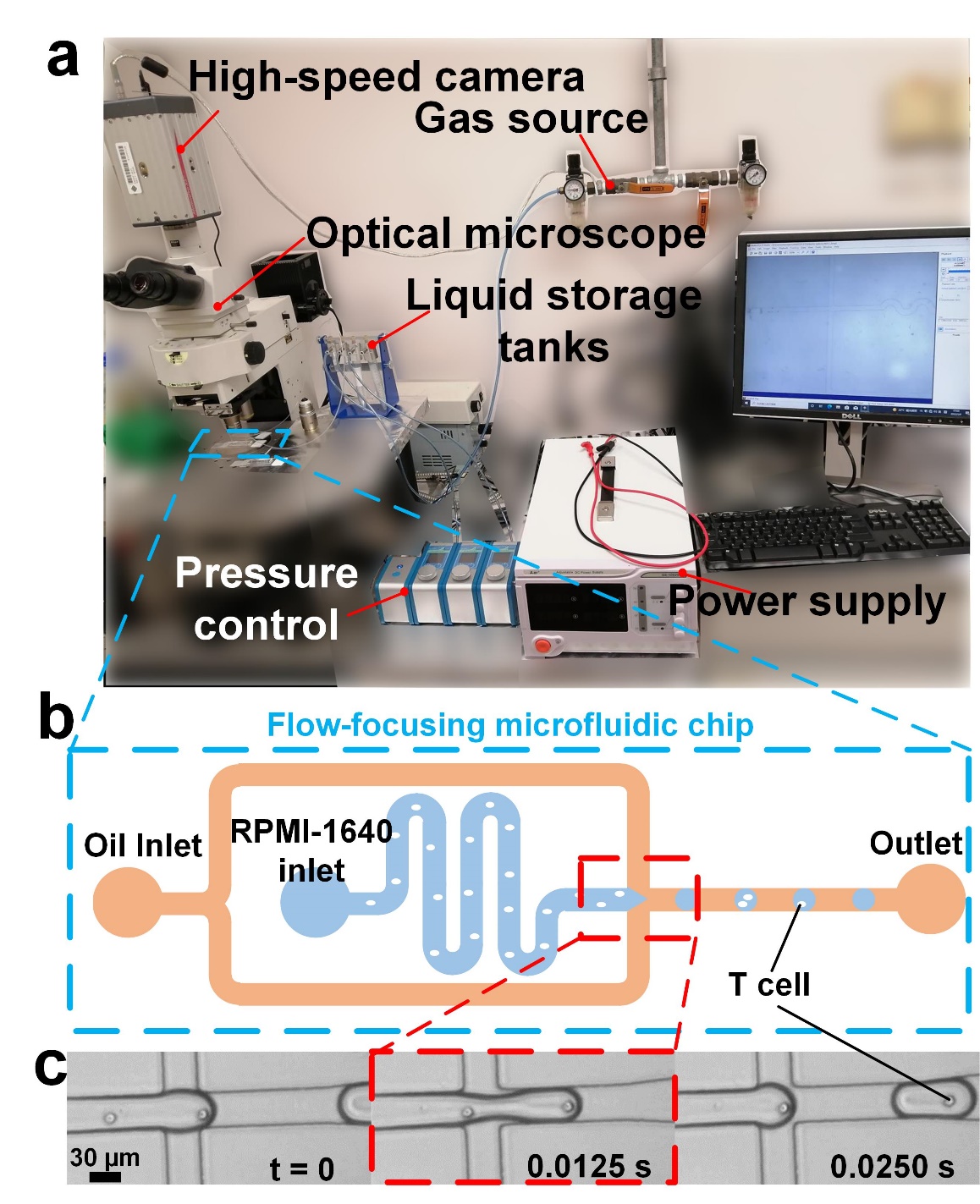  **Fig. S6. Flow-focusing microfluidic system for generating single-cell encapsulated droplets. a** Photographic view of the practical system. **b** Schematic view of the flow-focusing microfluidic chip with two inlets and one outlet. **c** Magnified view of the single-cell encapsulation process at the cross-junction area of the microfluidic chip. |
| --- |

**Note 4**

**Properties of fluids utilized in microfluidic droplets**

Table S2. Material properties for generating oil-water-oil double emulsions (DEs).

| Phases | Materials | Viscosity (Pa‧s) | Density (kg/m^3^) |
| --- | --- | --- | --- |
| Inner | Silicone oil | 0.001 | 963 |
| Middle | SDS-water solution (wt 3.34%) | 0.001 | 998 |
| Outer | Silicone oil | 0.001 | 963 |

Table S3. Parameters of the continuous and discrete phases for generating SDs.

| Phases | Material | Viscosity (Pa‧s) | Density (kg/m^3^) |
| --- | --- | --- | --- |
| Continuous | Silicone oil | 0.5 | 963 |
| Discrete | SDS-water solution  (wt 2.53%, 2.80%) | 0.001 | 998 |

**Note 5**

**Characterization of the nano-berries-laden liposomal DEs**

| 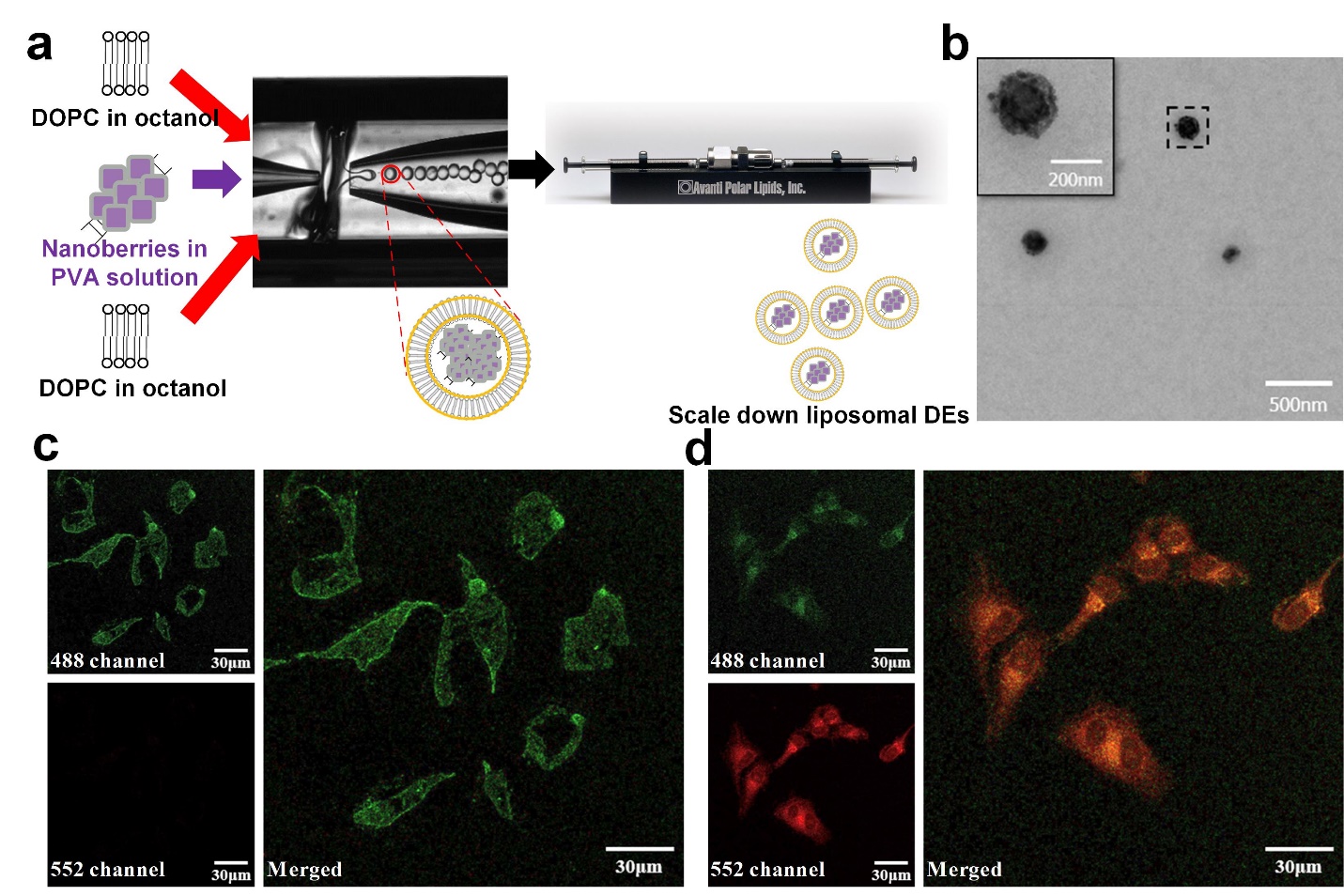  **Fig. S7. nano-berries-laden liposomal DE characterization relation.** **a** Scaled-down process before TEM characterization. **b** TEM images for the liposomal DE after being scaled-down. **c** Cellular uptake of nano-berries alone by HELA under confocal fluorescent microscope and **d** Cellular uptake of nano-berries encapsulated in the liposome. |
| --- |

As shown in Fig. S7a, the nano-berry-encapsulated liposome was synthesized through double emulsion in capillary-based microfluidics. The resulting DE droplets were then passed through a 100 nm-pore-sized extruder to further reduce the liposome size for enhanced cellular uptake. Fig. S7b presents the TEM image of the liposomal DEs after being scaled-down. Evaluation of cellular uptake was performed using nano-berries labeled with the FITC fluorescent tag. Comparing cellular uptake of nano-berries with and without liposome encapsulation (Figs. S7d and S7c, respectively), a significant increase was observed in the presence of the liposome, as indicated by fluorescence in the 488 channel. The FITC fluorescence of standalone nano-berries exhibited a concentrated region at the cell boundary, whereas that of nano-berries within the liposome showed increased aggregation within the cells, colocalizing with Nile red-labeled lipid in the 552 channel.

**Note 6**

**Identifications of phase change material (PCM) capsules**

Table S4. Physical properties of the PCM paraffin wax n-octadecane.

| State | Density (kg/m^3^) | Thermal conductivity  (W/m⋅K) | Specific heat  (kJ/(kg⋅K)) | Latent heat  (kJ/kg) | Phase-transition temperature (°C) |
| --- | --- | --- | --- | --- | --- |
| Solid | 880 | 0.21 | 2.90 | 178.5 (melting) | 27-28 |
| Liquid | 763.8 | 0.20 | 2.11 | 181.9 (solidification) |

Table S4 lists some significant properties of the utilized paraffin wax adopted for encapsulation. Additionally, Fig. S8 provides multi-scale SEM images of the morphological structure of a single empty capsule at an enlarged magnification. This highlights the dense solid capsule shell, which effectively prevents paraffin leakage.

| 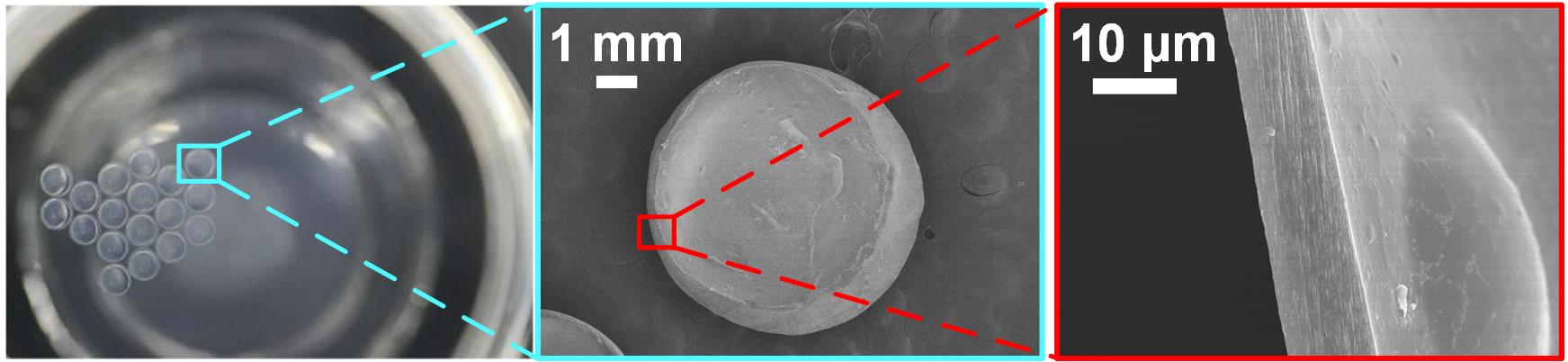  **Fig. S8. The internal structure of the PCM micro-capsules, as captured by SEM.** |
| --- |

**Note 7**

**Preliminary study before experimentally producing single-cell encapsulated droplets**

| 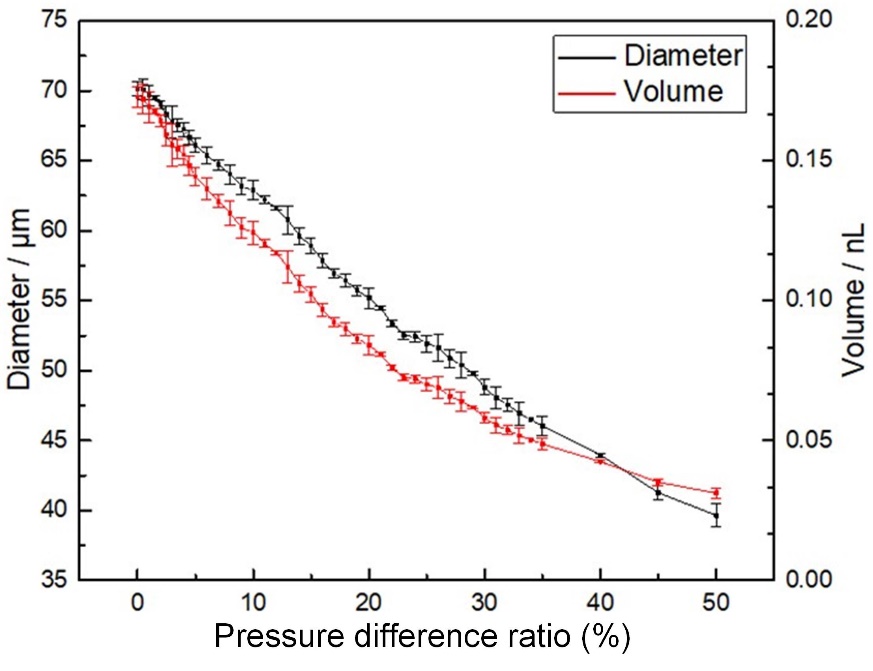  **Fig. S9. Droplet Size versus pressure difference ratio at the pressure of discrete phase of 120 mbar.** The droplet sizes were obtained by the Deformable DETR or edge detection algorithm. |
| --- |

Prior to generating the single-cell encapsulated droplets, droplet generation experiments were conducted to find a proper operating condition. Fig. S9 summarizes the droplet size relationship with the pressure difference ratio which is calculated by (*P_cont_* - *P_disp_*) / *P_disp_* (*P_disp_* = 120 mbar). The size of the droplet showed an inverse linear proportion to the pressure of the continuous phase. After obtaining the droplet size, we attained the droplet volume shown in the right axis of Fig. S9. Therefore, the number of droplets from 1 mL of discrete phase can be calculated accordingly. We selected the operating condition of *P_cont_* = *P_disp_* = 120 mbar to obtain droplets with larger sizes, which means the number of droplets from 1 mL of discrete phase can be minimized. Hence, the consumption of the purchased T cell can be minimized.

During the encapsulation of the cells into droplets, the possibility of a number of cells within one droplet conforms to Poisson’s distribution^6^ at low concentration:

| $P(n)=\frac{\lambda^{n}e^{-\lambda}}{n!}$, | (S9) |
| --- | --- |

where *n* is the number of cells in the droplet, $P(n)$ is the possibility of the droplet encapsulated with *n* cell, and $\lambda$ is the total cell-to-droplet ratio within 1 mL of sample. Fig. S10 plots the possibility distributions of droplets with *n* cell encapsulated with two values of $\lambda$ (0.5 and 0.3). Given the preference for a higher proportion of single-cell encapsulation in droplets and a lower proportion of multiple-cell encapsulation, the cell-to-droplet ratio was optimized to 0.5 cells per droplet. This corresponds to a cell concentration of 5×10^5^ cells per milliliter. The inserts in Fig. S10 displays the real cell encapsulations via the selected parameters above, showing the results are subject to the expected outcomes from Poisson’s distribution theory.

| 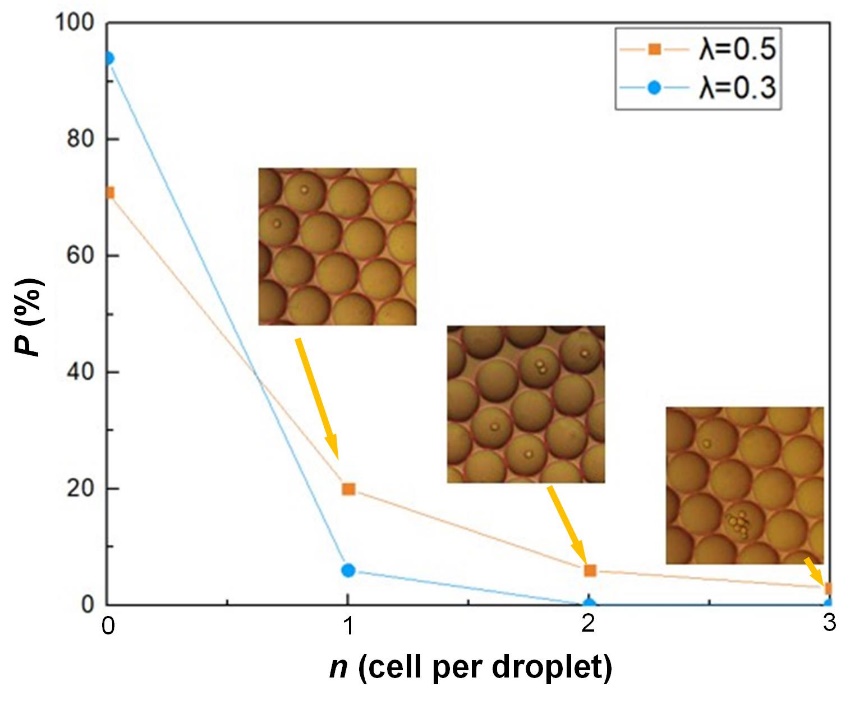  **Fig. S10. Possibility distribution of droplets with *n* cell encapsulated based on Poisson’s law.** |
| --- |

**Note 8**

**Additional detailed data presentations for droplets presented in Fig. 3**

Table S5. Detailed data in Fig. 3a ~ e.

| Sources | Parameters | Sample size | Average | Variance | min | Q1 | Median | Q3 | max | outlier |
| --- | --- | --- | --- | --- | --- | --- | --- | --- | --- | --- |
| Fig. 3a | *D_o_* (mm) | 16 | 0.2845 | 2.0300E-05 | 0.2767 | 0.2815 | 0.2847 | 0.2868 | 0.2951 | 0.2951 |
|  | *D_i_* (mm) | 16 | 0.1575 | 1.8603E-06 | 0.1557 | 0.1563 | 0.1571 | 0.1587 | 0.1596 | - |
|  | $\delta$ (%) | 16 | 55.35 | 1.0214 | 52.80 | 54.77 | 55.22 | 55.93 | 57.43 | 52.80 |
|  | $\tau$ (%) | 16 | 98.41 | 0.3515 | 97.44 | 98.17 | 98.45 | 98.70 | 99.49 | - |
| Fig. 3b | *D_o_* (mm) | 43 | 0.04272 | 8.3121E-07 | 0.04080 | 0.04242 | 0.04262 | 0.04312 | 0.04510 | 0.0451 |
|  | *D_i_* (mm) | 43 | 0.02953 | 6.7659E-07 | 0.02856 | 0.02893 | 0.02952 | 0.03023 | 0.03130 | - |
|  | $\delta$ (%) | 43 | 69.19 | 3.0448 | 65.43 | 68.19 | 69.20 | 69.93 | 74.53 | 65.43, 74.53 |
|  | $\tau$ (%) | 43 | 97.88 | 1.9314 | 92.88 | 97.06 | 98.24 | 98.99 | 99.51 | 92.88 |
| Fig. 3c | *D_o_* (mm) | 8 | 0.2826 | 1.8206E-03 | 0.2082 | 0.2546 | 0.2901 | 0.3164 | 0.3307 | - |
|  | *D_i_* (mm) | 8 | 0.1266 | 1.2582E-04 | 0.1095 | 0.1203 | 0.1257 | 0.1319 | 0.1478 | - |
|  | $\delta$ (%) | 8 | 45.5 | 41.2612 | 40.68 | 40.72 | 44.24 | 46.64 | 60.12 | 60.12 |
|  | $\tau$ (%) | 8 | 86.27 | 94.5114 | 75.13 | 77.19 | 85.85 | 95.80 | 97.34 | - |
| Fig. 3d | *D_o_* (mm) | 4 | 0.4679 | 5.3604E-05 | 0.4619 | 0.4623 | 0.4663 | 0.4735 | 0.4776 | - |
|  | *D_i_* (mm) | 4 | 0.3406 | 2.5433E-04 | 0.3223 | 0.3283 | 0.3404 | 0.3530 | 0.3593 | - |
|  | $\delta$ (%) | 4 | 72.85 | 19.8669 | 67.48 | 69.34 | 73.13 | 76.36 | 77.66 | - |
|  | $\tau$ (%) | 4 | 95.04 | 1.1461 | 93.97 | 94.15 | 94.96 | 95.93 | 96.25 | - |
| Fig. 3e | *D_o_* (mm) | 18 | 0.1581 | 9.3502E-05 | 0.1233 | 0.1546 | 0.1606 | 0.1635 | 0.1663 | 0.1230 |
|  | *D_i_* (mm) | 58 | 0.07145 | 3.5603E-05 | 0.04485 | 0.06743 | 0.07272 | 0.07502 | 0.08230 | 0.04480, 0.05500 |
|  | $\eta$ (%) | 18 | 29.1 | - | 18.1 | - | - | - | 36.1 | - |
|  | *n* | 58 | 3.22 | - | 2 | - | - | - | 4 | - |

Table S6. Statistical measures for microfluidic droplets marked by dotted orange in Figs. 3f, 3g, 3h and 3i.

| Sources | Parameters | Sample size | Average | Variance | Min | Q1 | Median | Q3 | Max | Outlier |
| --- | --- | --- | --- | --- | --- | --- | --- | --- | --- | --- |
| Fig. 3f | *D_o_* (mm) | 3 | 0.500 | 2.38e-5 | 0.495 | - | - | - | 0.504 | - |
|  | *D_i_* (mm) | 9 | 0.169 | 7.00e-4 | 0.139 | - | - | - | 0.207 | - |
|  | $\eta$ (%) | 3 | 12.49 | 5.20e-5 | 11.76 | - | - | - | 13.21 | - |
|  | *n* | 3 | 3 | 0 | 3 | - | - | - | 3 | - |
| Fig. 3g | *D_o_* (mm) | 19 | 0.457 | 5.01e-4 | 0.416 | 0.435 | 0.467 | 0.475 | 0.484 | - |
|  | *D_i_* (mm) | 19 | 0.351 | 9.49e-5 | 0.327 | 0.347 | 0.353 | 0.358 | 0.364 | 0.327, 0.331 |
|  | $\delta$ (%) | 19 | 76.94 | 9.16e-4 | 72.03 | 74.65 | 76.82 | 78.42 | 83.96 | - |
|  | $\tau$ (%) | 19 | 97.13 | 5.77e-4 | 92.61 | 95.00 | 98.20 | 99.22 | 99.84 | - |
| Fig. 3h | *D_o_* (mm) | 17 | 0.0479 | 1.91e-4 | 0.0299 | 0.0392 | 0.0450 | 0.0532 | 0.0824 | 0.0824 |
|  | *D_i_* (mm) | 17 | 0.0361 | 9.43e-5 | 0.0227 | 0.0290 | 0.0339 | 0.0415 | 0.0595 | - |
|  | *D_s_* (mm) | 2 | 0.0245 | 3.93e-7 | 0.0240 | - | - | - | 0.0249 | - |
|  | $\delta$ (%) | 17 | 75.61 | 8.06 e-4 | 70.42 | 73.40 | 75.46 | 78.03 | 80.00 | - |
|  | $\tau$ (%) | 17 | 97.09 | 3.60 e-4 | 92.66 | 95.98 | 97.49 | 98.41 | 99.88 | - |
| Fig. 3i | *D_o_* (mm) | 17 | 0.170 | 1.96e-5 | 0.158 | 0.168 | 0.170 | 0.173 | 0.177 | 0.158 |
|  | *D_i_* (mm) | 17 | 0.112 | 2.28e-6 | 0.110 | 0.110 | 0.112 | 0.113 | 0.116 | - |
|  | $\delta$ (%) | 17 | 65.86 | 2.98 e-4 | 62.39 | 64.53 | 66.00 | 67.00 | 70.02 | - |
|  | $\tau$ (%) | 17 | 97.64 | 5.05 e-4 | 90.22 | 97.14 | 98.38 | 98.93 | 99.52 | 90.22 |

**Note 9**

**Modified Fig. 3i for machine identification**

| 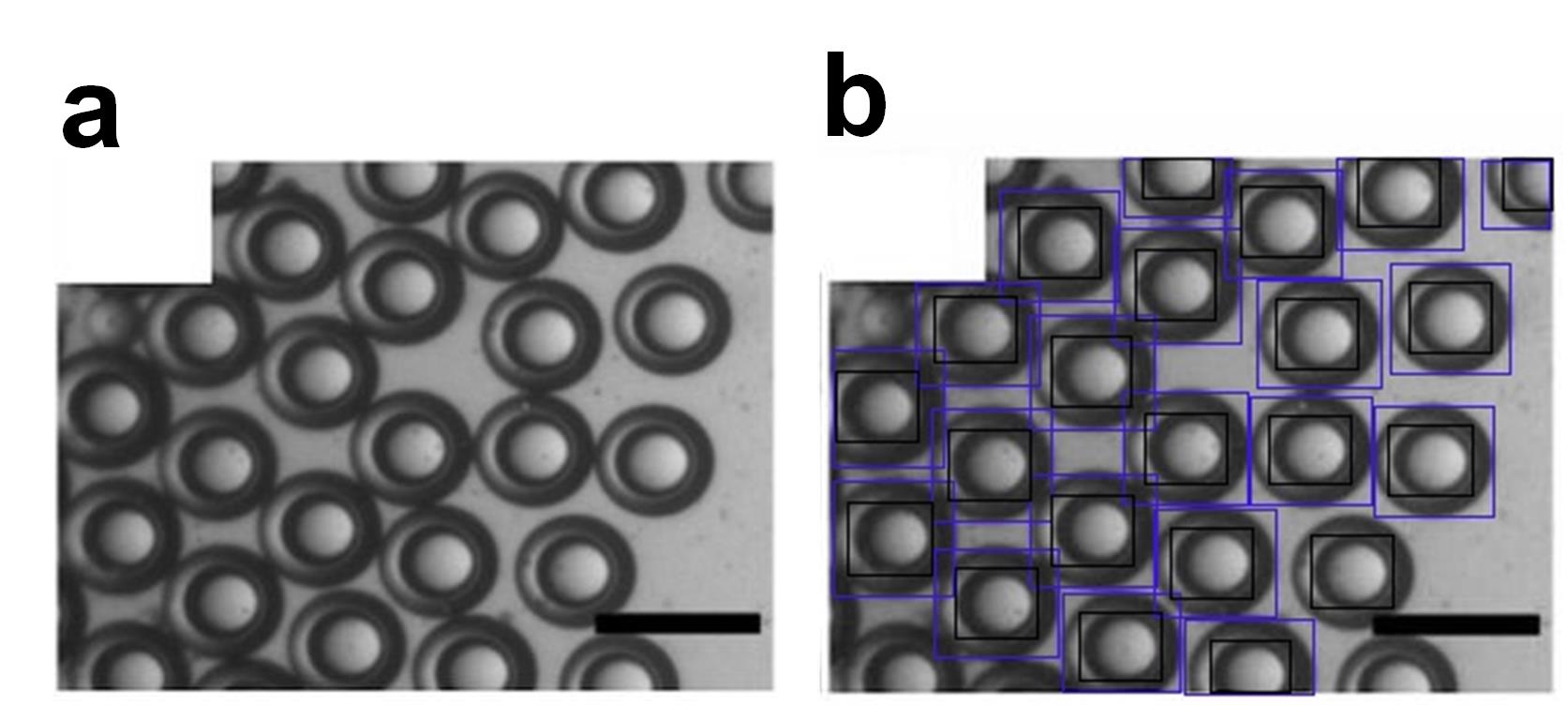  **Fig. S11. The corrected image without the “C” for machine visual identification (Adapted from Zhang et al.’s work^7^). a** Raw image**. b** input image after identification**.** |
| --- |

Different from Fig. 3i in the main paper, Fig. S11 here shows the image without the “C” for an accurate machine identification.

**Note 10**

**More image-based machine identification results**

| 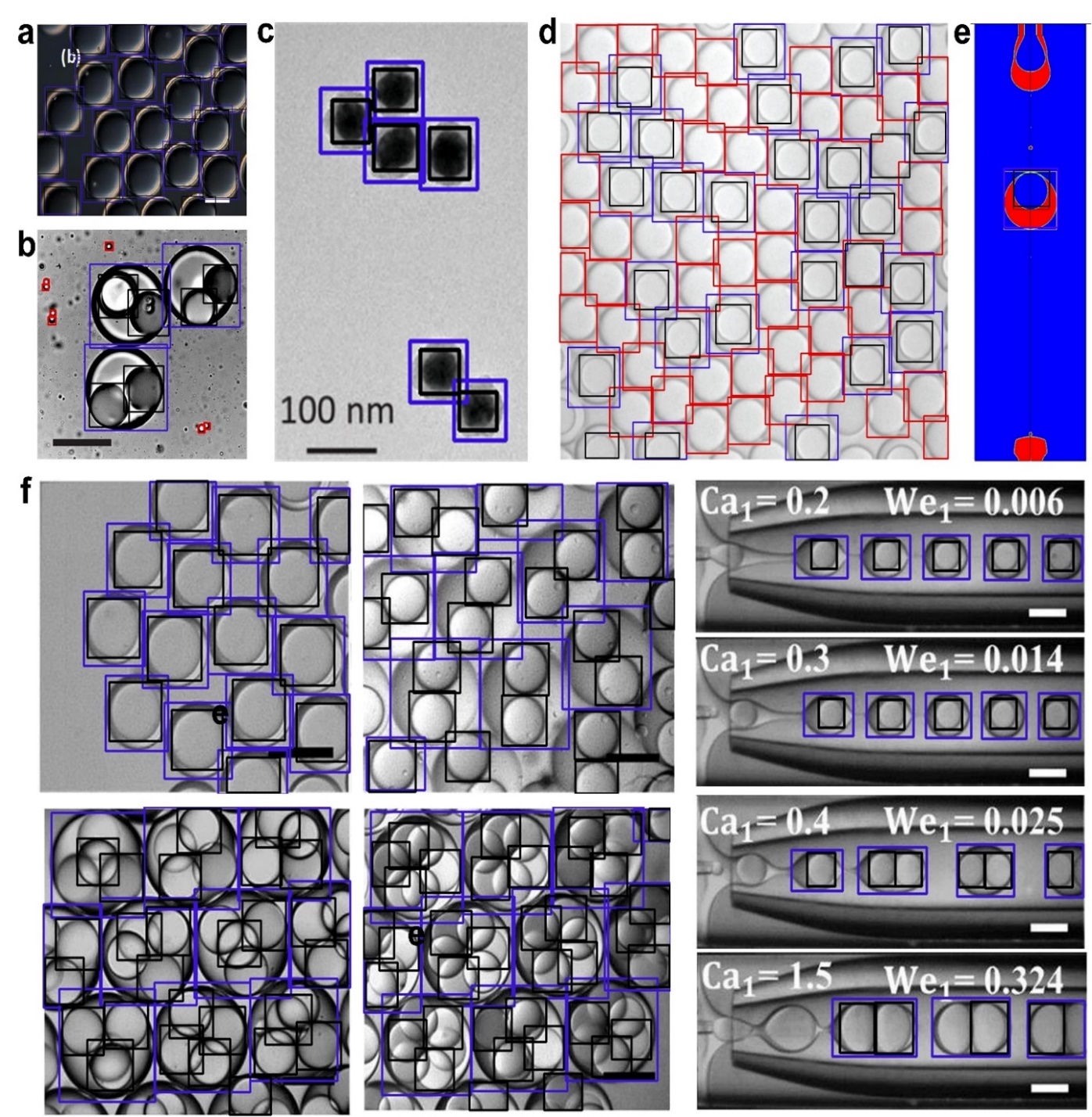  **Fig. S12. Extended machine identification results for a** Hughes’ work^8^, **b** Ma’s work^9^, **c** Liang’s work^10^, **d** Lashkaripour’s work^11^, **e** Numerical simulation of double emulsion production, and **f** Nabavi’s work^12^. |
| --- |

Fig. S12 demonstrates identification results of extended scenarios from more publications besides the identifications in Fig. 3 in the main paper. Besides the identifications of microfluidic droplets, Fig. S12c displays the identification of other core-shell structures in nanoscale. Fig. S12e demonstrates that the Deformable DETR is also capable of recognizing numerical simulation results of double emulsion generations.

**Note 11**

**Overall performance evaluation**

| 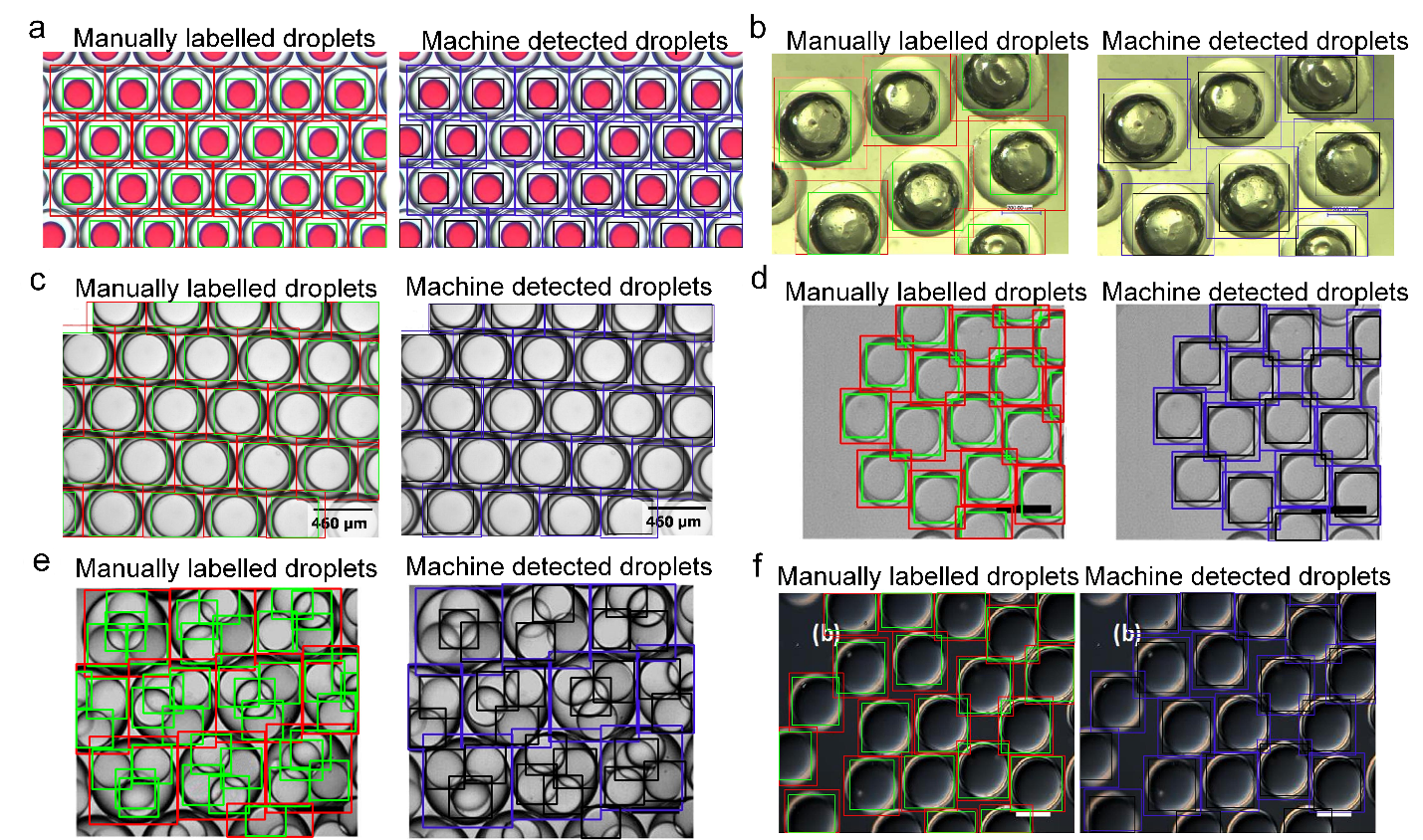  **Fig. S13. Comparisons between manually labelled droplets (ground truths) and machine detected droplets.** Original images from **a** Metter’s work^20^, **b** Fu’s work^21^. **c** Foster’s work^22^. **d** Nabavi’s work^12^, **e** Nabavi’s work^12^, and **f** Hughes’ work^8^. |
| --- |

Quantitative performance evaluation of the utilized Deformable DETR is presented Fig. S13 where the machine detected droplets will be compared with manually annotated ones (ground truths). The materials used for performance evaluation here are from Figs. 3 and S12. Several parameters such as length error $\delta_{l}$ (calculated by Eq. 1), area error $\delta_{A}$ (calculated by Eq. 2), intersection over union *IoU* (calculated by Eq. 3), accuracy *p* (calculated by Eq. S10) are considered to offer quantitative performance evaluations. *p* is a common metric used to evaluate the performance of a model. Accuracy measures the proportion of correctly classified samples out of the total number of samples^17^.

| $p=\frac{TP+TN}{TP+FP+FN+TN},$ | (S10) |
| --- | --- |

Where *TP* (True Positives) is the number of positive samples correctly identified as positive, *TN* (True Negatives) is the number of negative samples correctly identified as negative, *FP* (False Positives) is the number of negative samples incorrectly identified as positive, and *FN* (False Negatives) is the number of positive samples incorrectly identified as negative.

Table S7 lists the quantitative performance evaluation results based on the comparisons between manually labelled droplets (ground truths) and machine detected droplets as presented in Fig. S13. It shows that the average relative errors ($\delta_{l}$ and $\delta_{A}$) of the Deformable DETR can be controlled within 4% and the average precisions (*IoU* and *p*) are above as high as 93%.

Table S7. Overall performance evaluations

| Sources | $\delta_{l}$ | $\delta_{A}$ | *IoU* | *p* |
| --- | --- | --- | --- | --- |
| Fig. S13a | 2.88% | 2.69% | 95.17% | 96.08% |
| Fig. S13b | 3.02% | 2.53% | 94.92% | 87.50% |
| Fig. S13c | 3.44% | 3.31% | 94.07% | 100.00% |
| Fig. S13d | 3.85% | 3.17% | 93.44% | 92.45% |
| Fig. S13e | 3.07% | 2.83% | 94.41% | 97.14% |
| Fig. S13f | 3.80% | 4.92% | 93.40% | 89.11% |
| Average | 3.34% | 3.24% | 94.24% | 93.71% |

**Note 12**

**Application demonstrations of using the Deformable DETR for microfluidic droplets size characterization**

| 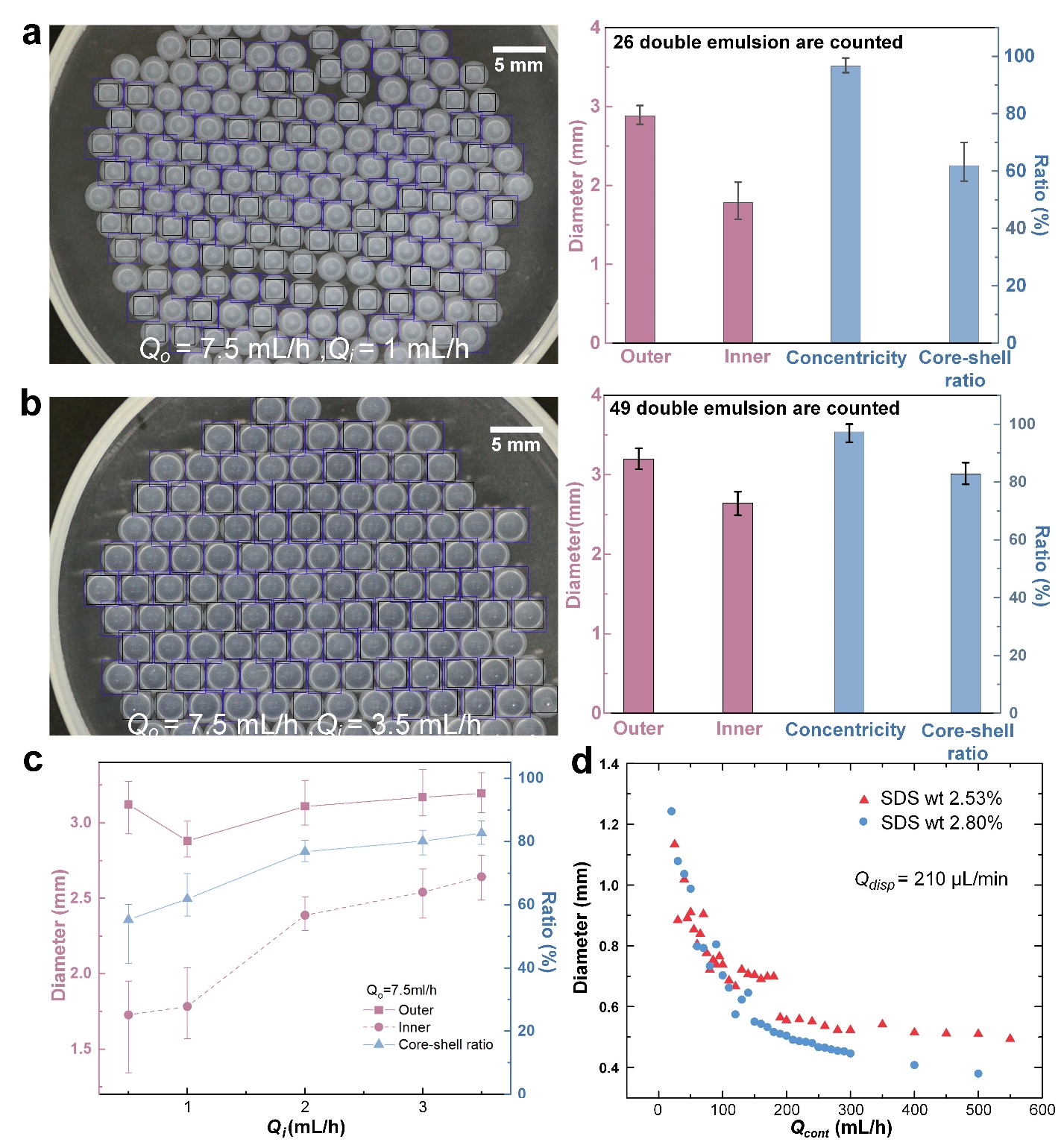  **Fig. S14. Size characterization using the Deformable DETR for microfluidic droplets from our own experiments. a** Identification for an array of PCM capsules with *Q_o_* = 7.5 mL/h and *Q_i_* = 1 mL/h and their size characterization. **b** Identification for an array of PCM capsules with *Q_o_* = 7.5 mL/h and *Q_i_* = 3.5 mL/h and their size characterization. **c** Statistical analysis of the PCM capsules for various flow rate combinations. **d** SD droplet diameter under various *Q_cont_* with a fixed *Q_disp_* (210 µL/min). |
| --- |

Fig. S14 presents data related to the characterization of microfluidic droplets using our own microfluidic device. Figs. S14a ~ c represents the size characterization for PCM capsules generated from the microfluidic co-extrusion device in Fig. S5. Fig. S14d is the size characterization for SDs from the co-flowing microfluidic chip in Fig. S4. The left-hand image of Fig. S14a shows the machine identification results for a large array of PCM capsules produced with a specific flow rate combination (*Q_o_* = 7.5 mL/h and *Q_i_* = 1 mL/h), and the right-hand image provides statistical analysis of the PCM capsules’ characteristics including their outer and inner diameters, concentricity, and core-shell ratio based on 26 identified DE targets as shown in the left-hand image of Fig. S14a. Fig. S14b displays the identification result and statistical analysis of another array generated with a different flow rate combination (*Q_o_* = 7.5 mL/h and *Q_i_* = 3.5 mL/h). Fig. S14c summarizes the statistical information for PCM capsules generated from multiple flow rate combinations (with a fixed *Q_o_* of 7.5 mL/h) including the conditions in Figs. S14a and b. It shows the outer and inner diameters as well as core-shell ratio rises as the *Q_i_* increases. When *Q_i_* reaches its maximum value (3.5 mL/h) here, the PCM capsules’ properties also reach an optimal condition with a large core-shell ratio, meaning a relatively large latent heat for phase-change-based thermal regulation^13,14^.

Fig. S14d shows the SDs’ diameters under various *Q_cont_* at a fixed *Q_disp_* (SDS wt 2.53% and 2.80%). The plot indicates how changing *Q_cont_* affects the size of the SDs, with the blue dots representing discrete phase of the wt 2.80% SDS-water solution and the red triangles representing discrete phase of the wt 2.53% SDS-water solution. As shown in the plot, the SD diameter decreases as the *Q_cont_* increases. An increasing *Q_cont_* means a higher rupture force created by the continuous phase, producing smaller microfluidic droplets. Besides, under the same flow conditions, the SDs from the wt 2.80% SDS-water solution are commonly smaller than those from the wt 2.53% SDS-water solution. A decrease in the mass fraction of SDS leads to an increase in interfacial tension. Higher interfacial tension provides a stronger force to confine droplet formation, allowing the droplets to maintain a larger volume before breaking off^15^. The size distribution plotted here is subject to the basic SD production laws^15,16^ in the co-flowing microfluidic device.

In summary, using the Deformable DETR allows us to rapidly determine and characterize the sizes of microfluidic droplets including SDs and DEs, with less human labor.

**Note 13**

**Identification and calculation logics of the Deformable DETR**

| 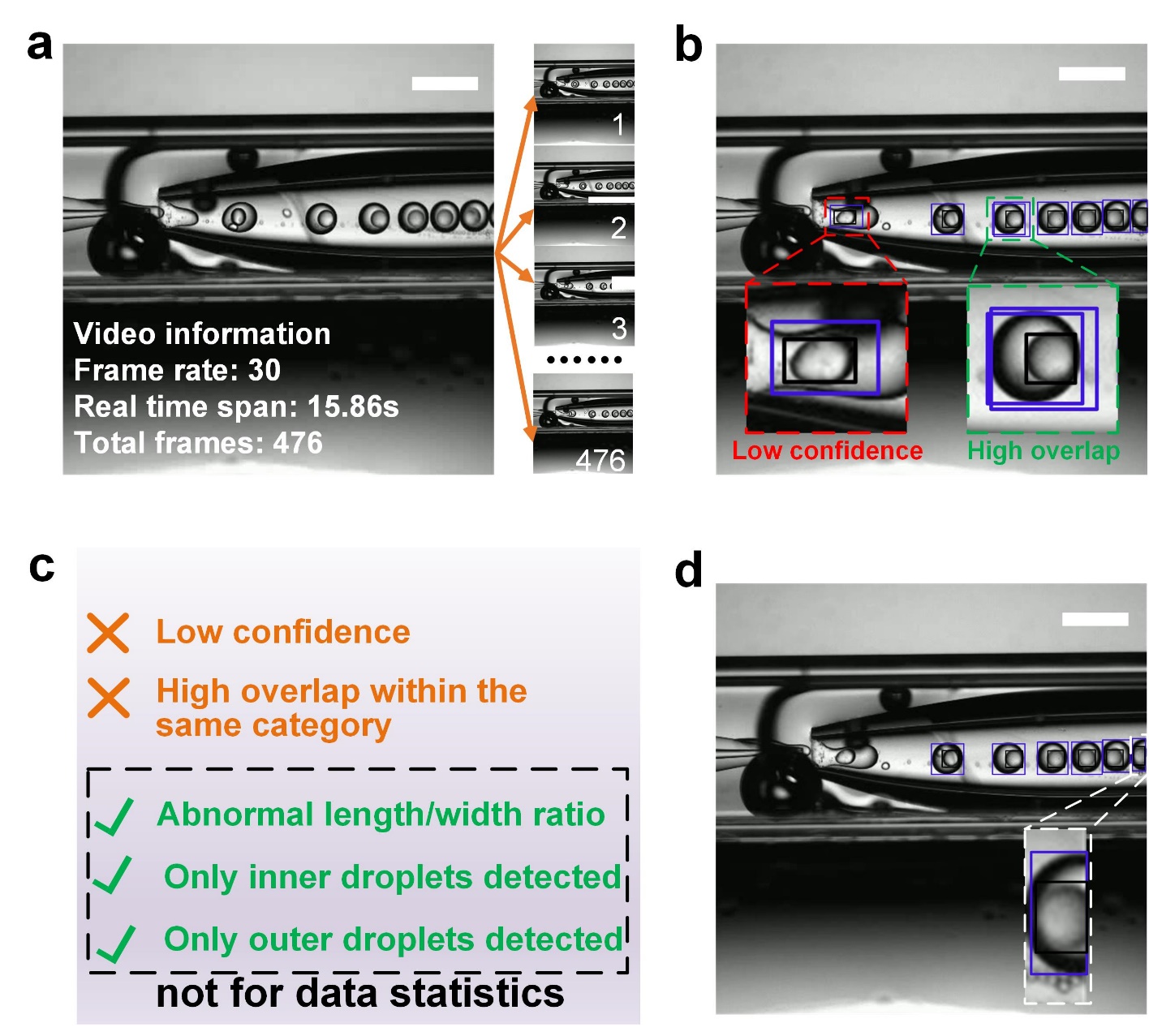  **Fig. S15. Singe-image-based microfluidic droplets identification. (Scale bar: 0.5 mm) a** The Movie used for identification is split into separate images, with each image representing a single frame of the movie. **b** Initial identification results. **c** Identified data quality control. **d** Final identification results. |
| --- |

The movie under identification is firstly decomposed into a set of frames. For example, as shown in Fig. S15a, the movie, which comes from our own experiments to obtain liposomal DEs, has a frame rate of 30 fps and a duration of 15.86 s (its real time span is also 15.86 s), resulting in 30 × 15.86 = 476 frames of images.

Next, the trained Deformable DETR model is used to detect droplets in each frame of the image sequentially to obtain initial detection results as shown in Fig. S15b. The magnified inserts show some unsatisfactory identification results. To further improve the precision of these detections, we use a data quality control strategy, as shown in Fig. S15c, to filter and handle certain special situations according to the following conditions: (1) Low confidence: In the initial detection results, confidence is an indicator used by the model to measure the reliability of its detections. Droplets with low confidence may be due to poor image quality or indistinct features of the droplet itself. To ensure the accuracy of the detection results, we exclude droplets with confidence below a set threshold (0.3). (2) High overlap of droplets of the same category: In a single frame, if identified multiple droplets (of the same category, either double emulsions or single emulsions) have high overlap (with a too high *IoU*), it suggests that the same droplet is detected multiple times. We set an overlap threshold (0.5), and when the *IoU* between two droplets exceeds 0.5, we retain only the detection result with the higher confidence and filter out other overlapping detections to avoid duplication. These two strategies are established to filter the bad identification results magnified in Fig. S15b. (3) Unreasonable length/width ratio of droplets: Droplets should be close to circular or elliptical in shape, so their length/width ratios should fall within a reasonable range. If the detected droplets are too flat or elongated, they may be false positives, or located at the edge of the image. Basically, we allow this identification, but we set a reasonable range for the length/width ratio to filter out detections that do not meet this criterion. We set a reasonable range for the aspect ratio to exclude detection results that do not meet this criterion from the data statistics. (4) Incomplete identification: If the droplet type is classified as a double emulsion, both inner and outer layers should be present. If the detection result includes only the inner droplet or the outer one, this might indicate an incomplete identification. We also allow this identification to be presented, but we will exclude incomplete detections from our data statistics. Fig. S15d shows the final identification results after applying the data quality control strategy. We can see decent identifications without the unsatisfactory detection displayed in Fig. S15b.

Based on the identification results, we track the droplets and employ frequency determination based on the movie-based identification as shown in Fig. S16. By comparing the position of droplets in the current frame with those in the previous frame, we calculate the *IoU* between frames to determine whether they represent the same droplet. As shown in Fig. S16a (i), we believe that the microfluidic droplets are the same one if *IoU* = *A_organge_*/*A_yellow_* > 0.2. After applying this algorithm, machine vision can identify which droplets across different frames are the same droplet, which lays the foundation for calculating droplet frequency. To obtain the frequency, we determine the number of droplets *N* traveling across a reference line in a certain time duration $\tau$. Therefore, the frequency can be obtained by $f=N/\tau$. Fig. S16a (ii), using the first three frames, demonstrates the counting process for a droplet crossing the reference line. In the first frame at the moment of *t*, the count is 0 since the focused droplet closest to the reference line has not yet reached it. In the second frame at the moment of *t* plus one unit of time step (represented by *t*+1), the front end of the droplet has passed the reference line but the whole droplet has not, so the count is *b*(*t*+1) / *w*(*t*+1), where *w* (*t*+1) represents the detected droplet length at this moment and *b*(*t*+1) is the length by which the front end of the droplet crosses the reference line. In the second frame at the moment of *t*+2, the rear end of the focused droplet has just touched the reference line, so the count is *b*(*t*+2) / *w*(*t*+2), which is approximately equal to 1. As time progresses and droplets continue to cross the reference line, the count accumulates according to the aforementioned rules shown in Fig. S16b. Therefore, during a certain time span $\tau$, it is possible to count N droplets crossing the reference line (where N can be an integer or a decimal number), and where the frequency can be acquired.

| 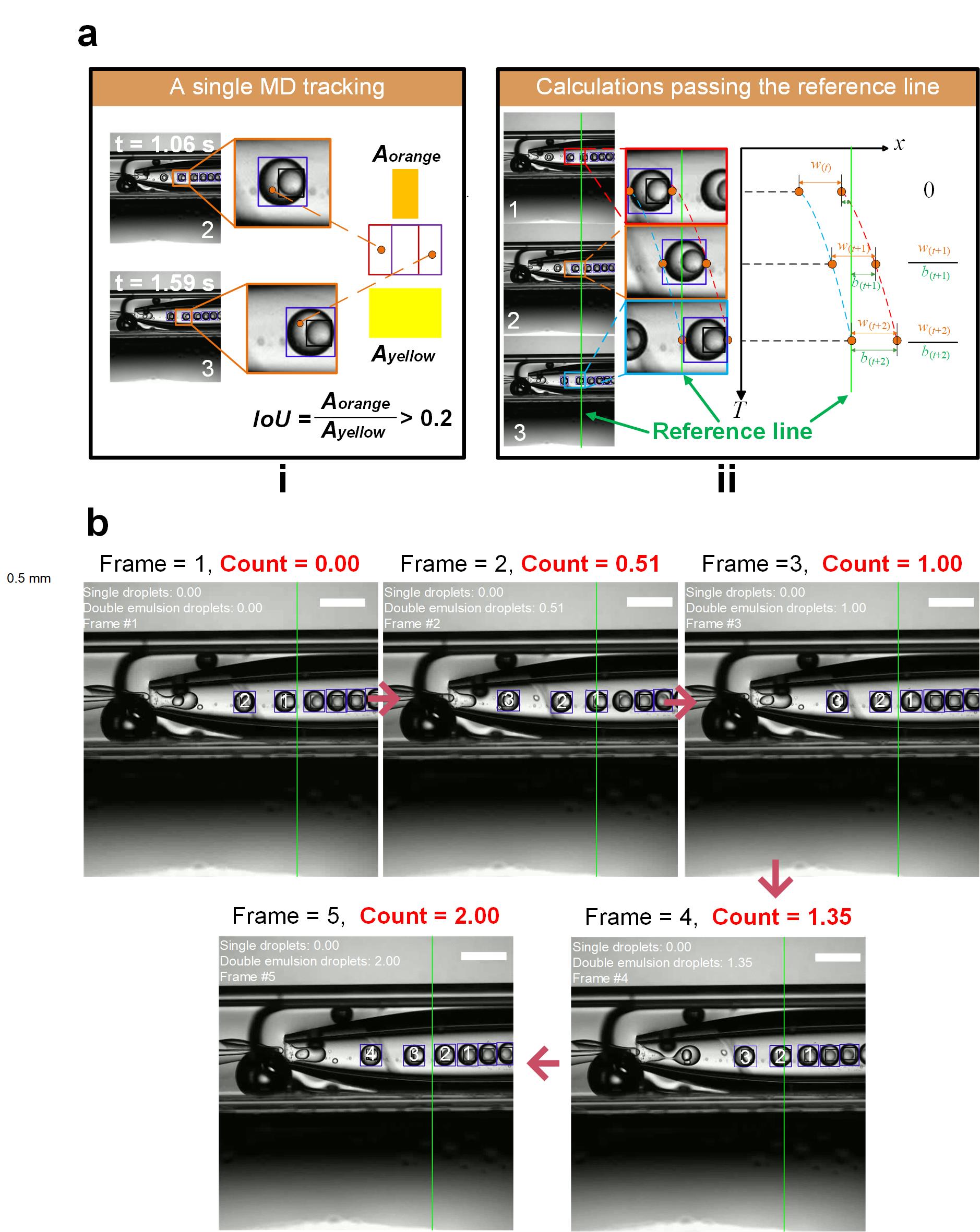  **Fig. S16. Detecting the trajectory of microfluidic droplets and frequency calculation. a** (i) Detecting the trajectory of a single microfluidic droplet, and (ii) calculations when a droplet passes across the reference line. **b** Frequency determination. |
| --- |

**Note 14**

**Erroneous identification**

As shown in Fig. S17, an identification error was found and magnified where the identified box (black one) for inner droplet was mistakenly enlarged. It happens mainly because the imaging quality at the edge is relatively poor. During the whole movie, there was a total of 5 DEs (out of 22) having such similar identification errors at the same location presented here. Such error identification results usually occur at the edge of an image due to insufficient image clarity. This is caused by the edge area being out of the camera focus.

| 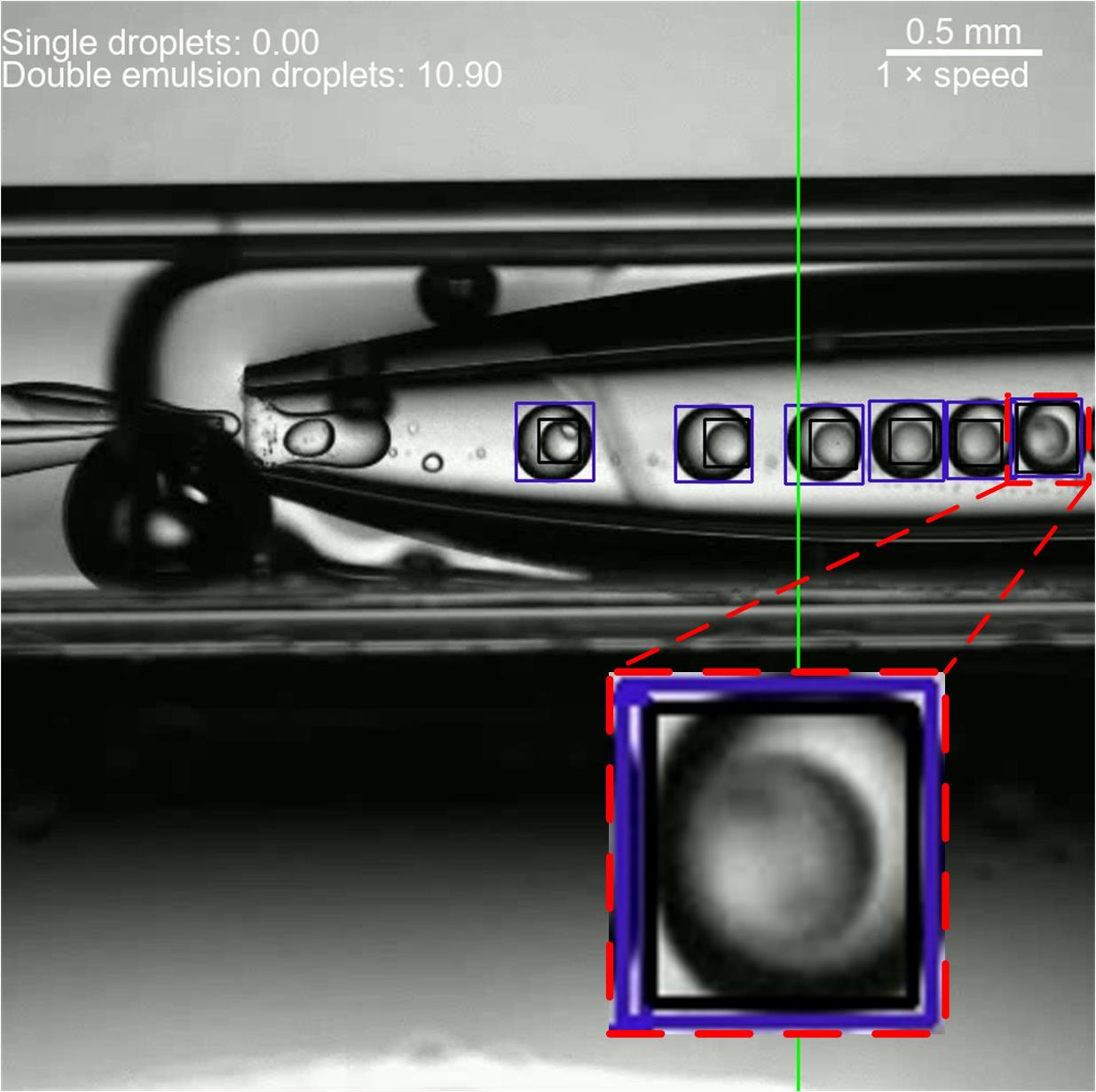  **Fig. S17. Abnormal identification demonstration.** |
| --- |

**Note 15**

**Image resolution enhancement method**

Bicubic interpolation is the method used to enhance the resolution of the target image in this paper. This method increases the resolution of digital images by adding new pixels and estimating their color values based on the surrounding pixels^18^. This technique aims to create a smoother and higher-quality image by generating additional pixel data. The process of bicubic interpolation: (1) Determine new pixel positions: calculate the corresponding position (x, y) of each new pixel in the original image. (2) Retrieve surrounding pixel values: centered at (x, y), select the 4 × 4 grid of surrounding pixels from the original image. These 16 pixels are used to calculate the new pixel value. (3) Calculate weights: compute the distance between the target pixel (x, y) and its 16 neighboring pixels; use a cubic interpolation function to determine the weight of each pixel. The closer a pixel is to the target, the greater its influence on the final value. (4) Horizontal interpolation: perform horizontal (x-direction) interpolation by applying the calculated weights to the pixel values along each row. This results in four intermediate values. (5) Vertical Interpolation: using the four intermediate values obtained from the horizontal interpolation, perform vertical (y-direction) interpolation to arrive at the final value for the new pixel. (6) By repeating these steps for every pixel in the new, larger image, we generate a higher-resolution version of the original. The bicubic interpolation method ensures a smooth transition between colors and reduces artifacts like jagged edges or blurriness that can occur when using simpler methods like nearest neighbor interpolation. The code for enhancing the image resolution in this paper is available at <https://github.com/MicrofluidicDroplets/Image-processing>.

**Note 16**

**Results of detection results for our own cell-involved experimental data**

| **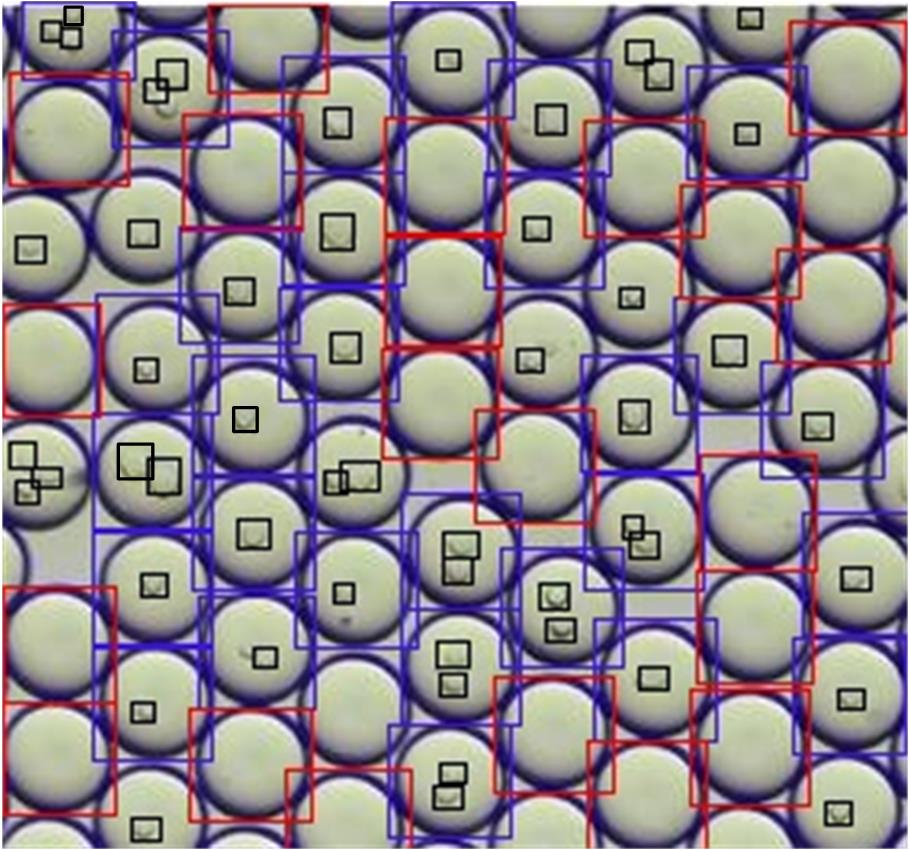**  **Fig. S18. Improved identification results of our own cell-involved experimental data** |
| --- |

**Note 17**

**Limitation demonstrations**

| **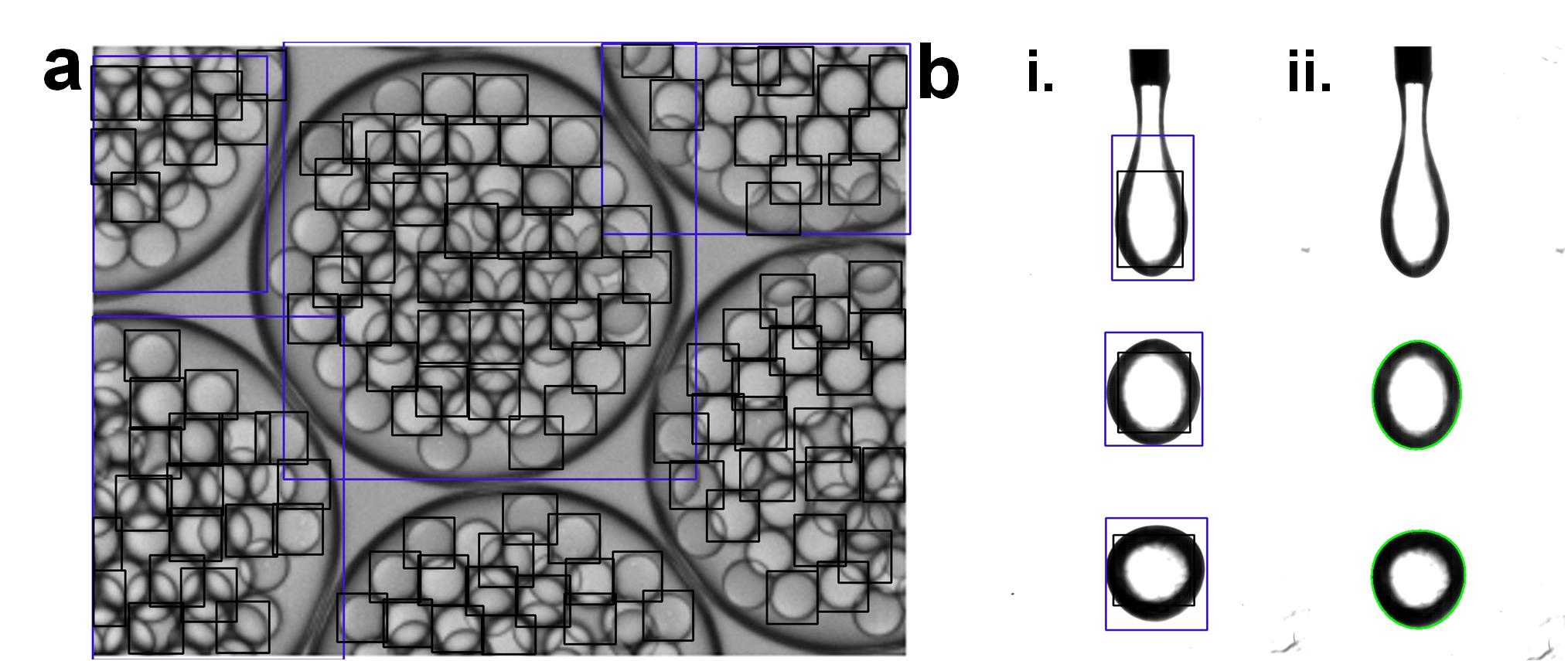**  **Fig. S19. Limitations of the Deformable DETR algorithm for microfluidic droplets identifications. a** Inner droplets identification failures (The original image is from Lashkaripour’s work^11^). **b** SDs identification using (i.) the proposed Deformable DETR and (ii.) edge detection algorithms. |
| --- |

Fig. S19 illustrates two typical limitations encountered with the Deformable DETR algorithm in the identification of microfluidic droplets. Fig. S19a demonstrates that Deformable DETR fails to recognize all the inner droplets within droplets exhibiting multiple cores. Droplets with multiple cores are expected to be distributed across different vertical planes, leading to instances where the imaging of all inner droplets cannot always be sufficiently clear. Upon reviewing Fig. S19a, it is observed that the majority of identification failures occur for inner droplets with indistinct edges, which is similar to Supplementary Note 11. This limitation originates from the inherent drawbacks associated with digital camera imaging and can potentially be mitigated through the application of image resolution enhancement techniques.

Fig. S19b (i) indicates that the Deformable DETR algorithm may erroneously classify SDs as DEs due to lighting conditions. The bright central part of SDs resembles the inner droplets of DEs, which explains why the algorithm might be misidentified. Alternatively, a basic edge detection algorithm^19^ can effectively address this limitation, as demonstrated in Fig. S19b (ii).

**References**

1. He, K., Zhang, X., Ren, S., & Sun, J. (2016). Deep residual learning for image recognition. In Proceedings of the IEEE conference on computer vision and pattern recognition, 770-778.
2. Liu, L., Ouyang, W., Wang, X., Fieguth, P., Chen, J., Liu, X., & Pietikäinen, M. (2020). Deep learning for generic object detection: A survey. Int. J. Comput. Vision, 128, 261-318.
3. Zhu, X., Su, W., Lu, L., Li, B., Wang, X., & Dai, J. Deformable DETR: Deformable transformers for end-to-end object detection. In Int. Conf. on Learning Representations (ICLR), 2021.
4. Vaswani, A., Shazeer, N., Parmar, N., Uszkoreit, J., Jones, L., Gomez, A. N., ... & Polosukhin, I. (2017). Attention is all you need. Advances in Neural Information Processing Systems, 30.
5. Ni, R., & Chau, Y. (2020). Nanoassembly of oligopeptides and DNA mimics the sequential disassembly of a spherical virus. Angew. Chem. Int. Edit., 59(9), 3578-3584.
6. Köster, S., Angile, F. E., Duan, H., Agresti, J. J., Wintner, A., Schmitz, C., ... & Weitz, D. A. (2008). Drop-based microfluidic devices for encapsulation of single cells. Lab on a Chip, 8(7), 1110-1115.
7. Zhang, H., Cui, W., Qu, X., Wu, H., Qu, L., Zhang, X., ... & Weitz, D. A. (2019). Photothermal-responsive nanosized hybrid polymersome as versatile therapeutics codelivery nanovehicle for effective tumor suppression. P. Natl. Acad. Sci. USA, 116, 7744-7749.
8. Hughes, E., Maan, A. A., Acquistapace, S., Burbidge, A., Johns, M. L., Gunes, D. Z., ... & Zick, K. (2013). Microfluidic preparation and self diffusion PFG-NMR analysis of monodisperse water-in-oil-in-water double emulsions. J. Colloid Interf. Sci., 389(1), 147-156.
9. Ma, S. (2019). Engineering inverse opals with enclosed voids via Bottom-up assembly of double emulsions Chem. Eng. Sci., 205, 414-419.
10. Liang, T., Li, Z., Bai, Y., & Yin, Y. (2024). Dichroic switching of core–shell plasmonic nanoparticles on reflective surfaces. Exploration, 4, 20210234.
11. Lashkaripour, A., McIntyre, D. P., Calhoun, S. G., Krauth, K., Densmore, D. M., & Fordyce, P. M. (2024). Design automation of microfluidic single and double emulsion droplets with machine learning. Nat. Commun., 15(1), 83.
12. Nabavi, S. A., Vladisavljević, G. T., & Manović, V. (2017). Mechanisms and control of single-step microfluidic generation of multi-core double emulsion droplets. Chemical Engineering Journal, 322, 140-148.
13. Wang, J. X., Qian, J., Wang, N., Zhang, H., Cao, X., Liu, F., & Hao, G. (2023). A scalable micro-encapsulated phase change material and liquid metal integrated composite for sustainable data center cooling. Renew. Energ., 213, 75-85.
14. Wang, J. X., Lai, H., Zhong, M., Liu, X., Chen, Y., & Yao, S. (2023). Design and Scalable Fabrication of Liquid Metal and Nano‐Sheet Graphene Hybrid Phase Change Materials for Thermal Management. Small Methods, 7(9), 2300139.
15. Lian, J., Luo, X., Huang, X., Wang, Y., Xu, Z., & Ruan, X. (2019). Investigation of microfluidic co-flow effects on step emulsification: Interfacial tension and flow velocities. Colloid. Surface A, 568, 381-390.
16. Wang, J. X., Qian, J., Wang, H., Sun, M., Wu, L., Zhong, M., ... & Mao, Y. (2024). Dual-directional small-sampling deep-learning modelling on co-flowing microfluidic droplet generation. Chem. Eng. J., 149467.
17. Bishop, C. M., & Nasrabadi, N. M. (2006). Pattern recognition and machine learning. New York: Springer.
18. Safinaz, S., & Kumar, A. R. (2017). VLSI realization of Lanczos interpolation for a generic video scaling algorithm. In 2017 IEEE International Conference on Recent Advances in Electronics and Communication Technology (ICRAECT), 17-23.
19. Wang, J. X., Cui, B., Salmean, C., Chen, X., Yan, X., Mao, Y., & Yao, S. (2024). Machine-assisted quantification of droplet boiling upon multiple solid materials. Nano Energy, 125, 109560.
20. Mettler, M. (2022). Controlled double emulsification process for encapsulation. https://secoya-tech.com/wp-content/uploads/2022/03/WP-GENERATION-OF-MICROCAPSULES-020322.pdf
21. Fu, Z., Su, L., Li, J., Yang, R., Zhang, Z., Liu, M., ... & Li, B. (2014). Elastic silicone encapsulation of n-hexadecyl bromide by microfluidic approach as novel microencapsulated phase change materials. Thermochimica Acta, 590, 24-29.
22. Foster, T., Dorfman, K. D., & Davis, H. T. (2010). Giant biocompatible and biodegradable PEG–PMCL vesicles and microcapsules by solvent evaporation from double emulsion droplets. J. Colloid Interf. Sci., 351, 140-150.

**Supplementary Movies**

**Movie 1.** Failure in double emulsion identification using edge detection algorithm.

**Movie 2.** Movie-based double emulsion identifications using Deformable DETR.

**Movie 3.** Extended identifications using Deformable DETR.

**Movie 4.** Guidance and operation demonstration of MDIA's machine identification functionality.

**Movie 5.** Guidance and operation demonstration of MDIA's transfer learning functionality.
